# Organoplatinum(II) Complexes Self-Assemble and Recognize AT-Rich Duplex DNA Sequences

**DOI:** 10.1021/acs.inorgchem.0c02648

**Published:** 2021-01-27

**Authors:** Ana Zamora, Erin Wachter, María Vera, David K. Heidary, Venancio Rodríguez, Enrique Ortega, Vanesa Fernández-Espín, Christoph Janiak, Edith C. Glazer, Giampaolo Barone, José Ruiz

**Affiliations:** ‡ Departamento de Química Inorgánica, Universidad de Murcia, and Biomedical Research Institute of Murcia (IMIB-Arrixaca), E-30071 Murcia, Spain; § Department of Chemistry, University of Kentucky 505 Rose Street, Lexington, Kentucky 40506, United States; ⊥ Departamento de Química Física, Universidad de Murcia, E-30071 Murcia, Spain; ∥ Institut für Anorganische Chemie und Strukturchemie, Heinrich-Heine-Universität Düsseldorf, D-40204 Düsseldorf, Germany; # Dipartimento di Scienze e Tecnologie Biologiche, Chimiche e Farmaceutiche (STEBICEF), Università di Palermo, 90128 Palermo, Italy

## Abstract

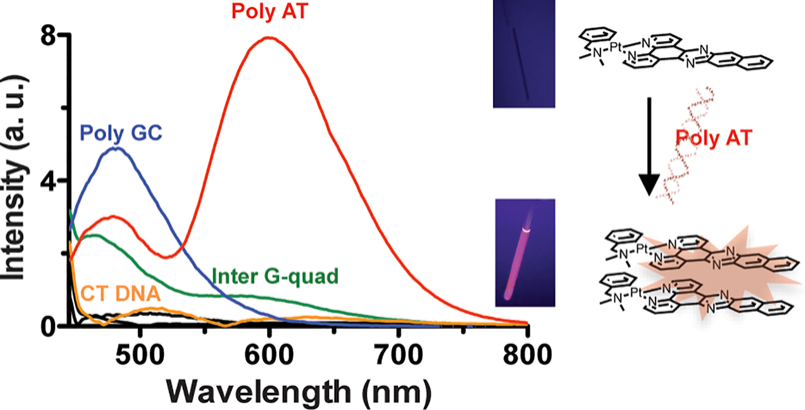

The specific recognition of AT-rich DNA sequences opens up the door to promising diagnostic and/or therapeutic strategies against gene-related diseases. Here, we demonstrate that amphiphilic Pt^II^ complexes of the type [Pt(dmba)(N∧N)]NO_3_ (dmba = *N*,*N*-dimethylbenzylamine-κ*N,* κ*C*; N∧N = dpq (**3**), dppz (**4**), and dppn (**5**)) recognize AT-rich oligonucleotides over other types of DNA, RNA, and model proteins. The crystal structure of **4** shows the presence of significant π-stacking interactions and a distorted coordination sphere of the d^8^ Pt^II^ atom. Complex **5**, containing the largest π-conjugated ligand, forms supramolecular assemblies at high concentrations under aqueous environment. However, its aggregation can be promoted in the presence of DNA at concentrations as low as 10 μM in a process that “turns on” its excimer emission around 600 nm. Viscometry, gel electrophoresis, and theoretical calculations demonstrate that **5** binds to minor groove when self-assembled, while the monomers of **3** and **4** intercalate into the DNA. The complexes also inhibit cancer cell growth with low-micromolar IC_50_ values in 2D tissue culture and suppress tumor growth in 3D tumor spheroids with a multicellular resistance (MCR) index comparable to that of cisplatin.

## Abstract 2

Cyclometalated Pt^II^ complexes containing π-conjugated ligands form supramolecular assemblies under aqueous environment, and DNA-induced aggregation occurs for the one containing the highest conjugated *N,N*-diimine ligand. The complexes recognize AT-rich DNA sequences over others in DNA, RNA, and proteins. Their DNA binding mode switches from intercalation to minor groove binding when self-assembled. The complexes suppress tumor growth in 3D tumor spheroids.

## Introduction

Fluorescence probes that target specific subcellular organelles and biomolecules, such as proteins or DNA, display enormous potential in biomedical applications ranging from diagnosis to therapy or a combination of the two.^1^ Clearly, DNA remains the most promising biological target for gene-related diseases such as cancer. New therapeutic strategies can be developed by controlling certain DNA functions, as well as elucidating complex and intertwined biological pathways in living cells.^2a^ This could be achieved by turning the expression of a gene either on or off, which requires DNA binding probes capable of recognizing specific sequences or base-pairs.^2a^ In addition, these nucleic acid–based approaches that rely on small molecules circumvent the problems associated with other forms of gene therapy and can effectively inhibit transcription or translation *in vitro*.^2b^ The interest in progressing toward selective therapies has resulted in the development of selective DNA binding agents, ranging from small molecules to large peptides.^2^ Among the small synthetic molecules, a highlight is metallointercalators of the type [ML_2_(dppz)]^*n*+^ (L = bpy or phen; bpy = 2,2′-bipyridine; phen = 1,10-phenanthroline; dppz = dipyrido-[3,2-*a*:2′,3′-*c*]phenazine). The incorporation of a transition metal not only imparts a positive charge into the dppz intercalator design but also offers other features such as luminescence (especially when M = Ru^II^)^3^ and/or cytotoxicity (M = Ir^III^ or Rh^III^).^4^ When incorporated into DNA-intercalating moieties, these dipyridophenazine centers are of particular interest because they confer light-switching properties in the presence of DNA.^3,5−8^

Typically, octahedral metallointercalators bind through an enantioselective intercalation mode between base-pairs, either to the major or minor groove, with a distribution of stacking orientations.^7^ As with simple intercalators that show only limited sequence preference, targeting coligands can be engineered into the octahedral complexes for selective detection of duplex DNA as well as different DNA sequences, such as mismatches and abasic sites.^6^ Notably, Ru-dppz complexes have been modified to target and stabilize guanosine-rich regions, allowing a remarkable visualization of G-quadruplex structures over duplex DNA.^8^ However, in contrast to oligonuclear complexes,^9^ mononuclear dppz complexes rarely target AT·TA steps.

Selective binding to purine–pyrimidine sequences using minor groove-binding molecules is considered a promising molecular recognition strategy since AT-rich regions play an essential role in a variety of nuclear activities, such as gene transcription, DNA replication, chromatin remodeling, and DNA repair.^10^ Minor groove binders interfere directly with gene transcription by inhibition of protein–DNA interactions, which has conferred upon them numerous biological activities such as antitumor, antiprotozoal, antiviral, and antibacterial properties.^2a,7^ Of particular note is the high affinity of *N*-methylpyrrole and *N*-methylimidazole-containing crescent-shaped polyamides for the AT-rich regions of the minor groove. The “*isohelicity*” of these molecules provides them with a molecular curvature that fits perfectly into the DNA minor groove concavity.^11^ Achieving this shape with metal complexes remains challenging, and therefore, targeting AT regions in the minor groove with an inorganic compound is not an easy task.

However, supramolecular chemistry affords new opportunities for the design of molecules with a predetermined shape, allowing for a specific fit and orientation. Favorable π–π stacking is known to lead to the formation of dimers or even higher order aggregates in solution, and in a controlled fashion (i.e., spherical, platelets, hexagonal plates, and sheet-like morphologies).^12^ More importantly, the self-assembly of the complexes under biologically relevant conditions has provided them with improved biological activities^13^ and photophysical properties.^14^ It is well-known that octahedral dppz complexes generally exhibits reversible aggregation in water, in spite of the large π-surface of this ligand. Consequently, it is frequently observed that aggregates of these complexes dissolve when studied at low concentrations.^15^ In contrast, aggregation of Pt^II^ complexes is promoted in solution due to their square-planar geometry, π–π and hydrophobic interactions of the ligands, as well as by Pt–Pt interactions at short distances (<3.5 Å).^13c,16^ These features make Pt^II^ complexes very sensitive toward microenvironmental changes, and the formation of supramolecular Pt^II^ assemblies can be induced in a controlled fashion in the presence of ordered counter-anions. This can also alter the photophysics of the complexes. For example, cationic alkynyl Pt^II^ terpyridine complexes self-assemble in the presence of certain nucleic acids and enzymes, and as a result, a low-energy metal–metal-to-ligand charge transfer (MMLCT) absorption band and a near-infrared (NIR) emission appear.^17^ These spectroscopic changes have allowed differentiation of specific molecules from their respective metabolic products that differ by a single structural feature. It also allows the real-time monitoring of the activities of the important enzymes, such as kinases and phosphatases, through differentiation of phosphorylated substrates.^17^

We prepared a series of amphiphilic organometallic Pt^II^ complexes of the type [Pt(dmba)(N∧N)]NO_3_ where the cyclometalating ligand (dmba = *N*,*N*-dimethylbenzylamine-κN,κC) remains as the C∧N backbone and the diimine ligand has been varied (N∧N = bpy (**1**), phen (**2**), dpq (**3**), dppz (**4**), and dppn (**5**); dpq = dipyrido[3,2-*d*:2′,3′-*f*]quinoxaline; dppn = benzo[*i*]dipyrido [3,2-*a*:2′,3′-*c*]phenazine). The bpy and phen complexes, with a smaller π-conjugated surface, were prepared for comparative purposes. The aggregation of **5** was studied in an aqueous environment, and their “light-switch” behavior was tested for different DNA sequences. The differences between the light-switch behavior of **3**–**5**have been correlated to a distinct DNA binding mode, demonstrated through electrophoretic mobility shift assay and viscometry, as well as TD-DFT and QM/MM calculations. Finally, their biological activity has been tested in both 2D tissue culture and 3D tumor spheroids. We describe here the sequence selective behavior, altered photophysics, and biological activity of these molecules.

## Results and Discussion

### Synthesis and Characterization

**Chart 1:**
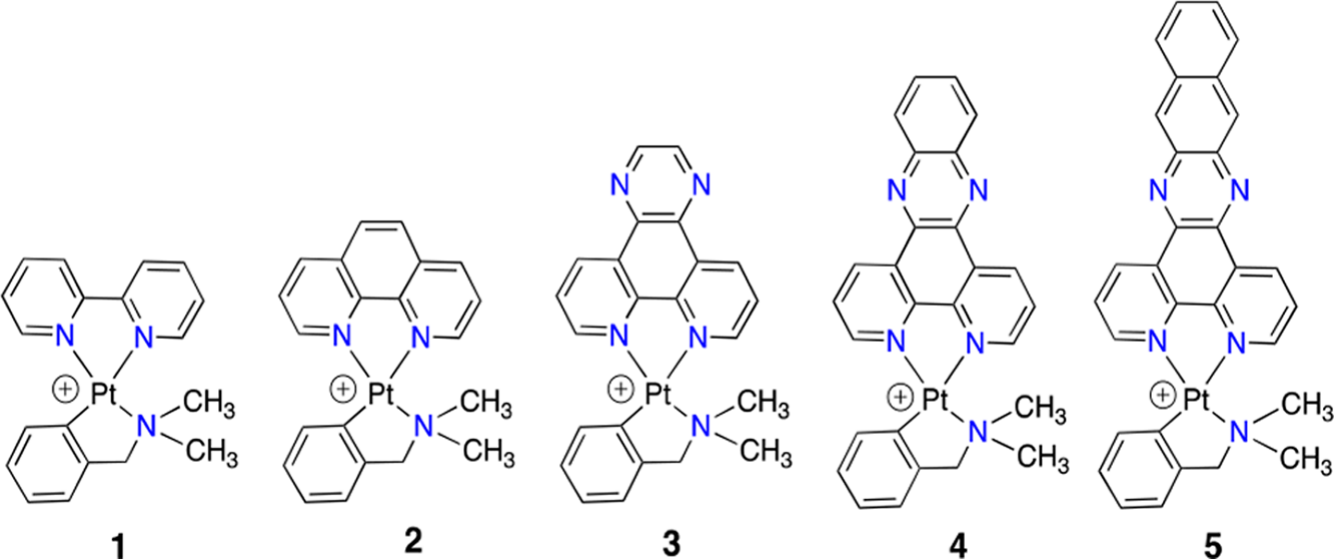
Structures of Pt^II^ Polypyridyl Complexes **1**–**5** in This Work

**Figure 1:**
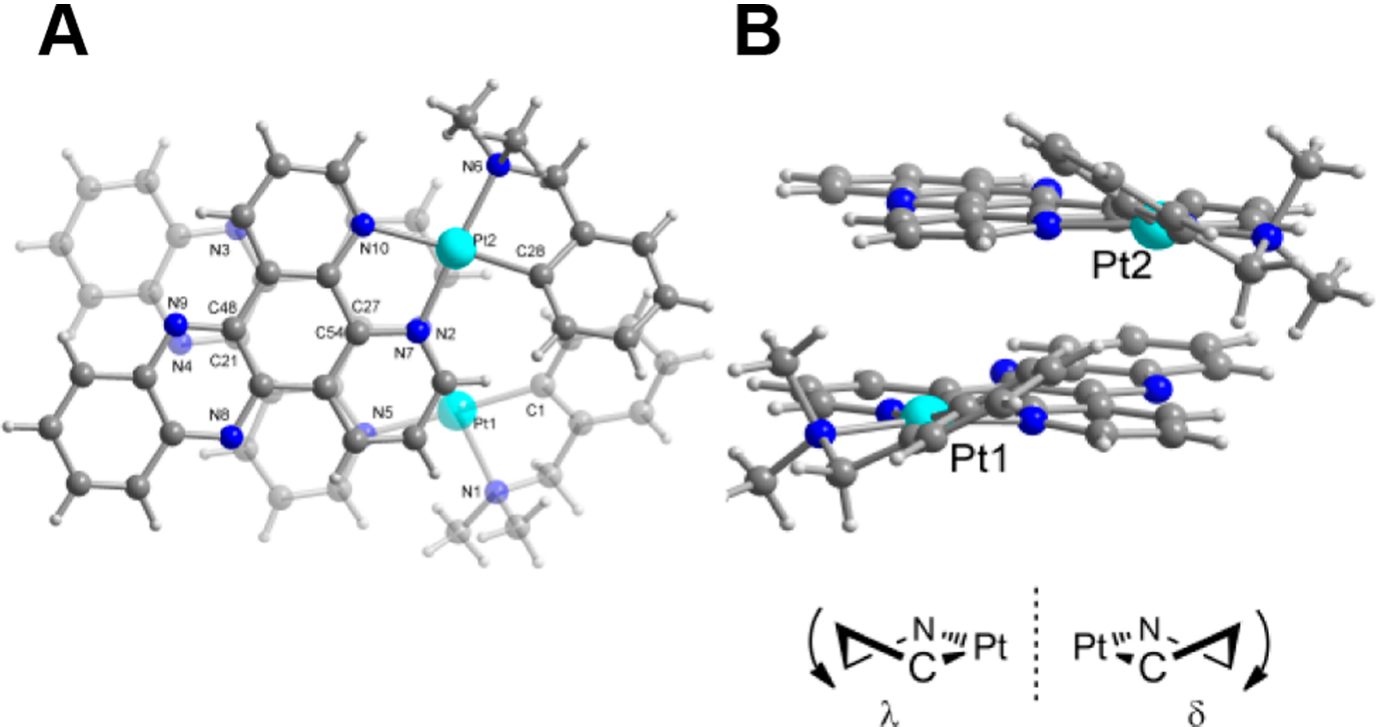
Top (A) and front (B) view of the two symmetry-independent cations of **4**. In the top view, the bottom molecule of Pt1 is depicted semitransparent for clarity.

Platinum complexes **1**–**5** (Chart 1), synthesized as their NO_3_^–^ salts to ensure high water solubility, were prepared by the chloride abstraction of the cyclometalated [Pt(dmba)(DMSO)Cl] precursor with a silver salt and the subsequent addition of the corresponding diimine ligand in a 1:1 molar ratio. All complexes were isolated in moderate to good yields (31–68%) and fully characterized by ^1^H, ^13^C and ^195^Pt NMR spectroscopy, HR-ESI-MS, elemental analysis, and RP-HPLC (Figures S1–S11). In addition, the structures of **2** and **4** were unambiguously confirmed by X-ray (see a detail discussion in the Supporting Information). Figure 1 depicts the ORTEP diagram of complex **4**. The crystal structure shows the presence of two symmetry-independent cations organized by intermolecular π–π interactions and reveals a non-planar coordination sphere of the d^8^ Pt^II^ atom. A similar distortion of the square-planar geometry was previously observed in cyclopalladated complexes containing secondary benzylamines.^18^ The five-membered platinum-*N,N*-dimethylbenzylamine chelate ring assumes a chiral conformation (λ at Pt1 and δ at Pt2, Figure 1B). Often, these λ/δ enantiomeric conformations of a five-membered chelate ring have a low energy barrier for interconversion through a planar transition state, and are only observed in the solid state. Complex **2** crystallizes in the *P*1̅ space group, and the packing in the structure is also organized by intermolecular π–π interactions. The main difference is that there is only one symmetry-independent molecule in **2**, i.e., the λ enantiomer. No Pt–Pt interactions are observed in either case, since the closest Pt–Pt distance is ∼5.3 Å. Therefore, the metal complexes have the necessary properties that render them especially suitable for reversible self-assembly.

### Aggregation Behavior

In contrast to complex **5**, which had a very limited solubility in aqueous media (*S* = 191 μM), **1**–**4** dissolved in water at concentrations up to 1 mM. This observation pointed toward the aggregation properties of **5**, revealing that hydrophobic π-stacking interactions are likely to occur at high concentrations in an aqueous environment. In fact, the tendency of complex **5** to aggregate in solution was observed by ^1^H NMR when increasing the concentration and at different compositions of mixtures of DMSO-[*d*_6_] and D_2_O. As shown in Figure S13, an upfield shift of the proton signals was observed with increasing concentration, while the signals become poorly resolved as the percentage of D_2_O increases from 0 to 10%, and virtually disappear at higher percentages. This suggests that the size of the π surface of the polypyridyl ligand is essential for their self-assembly, as it provides an additional hydrophobic interaction. Nevertheless, it is generally accepted that the aggregation of dppz-type complexes is reversible and disaggregation occurs at low complex concentrations. Thus, to provide further insights into the self-assembly process of the complexes, the molecular interactions of **3**–**5** were studied by evaluating the absorption and emission properties in Tris–HCl buffer (5 mM, pH 7.0). The UV/vis absorption spectra of **3**–**5** are dominated by a series of ligand-centered absorption bands in the range 200–300 nm and a lowest energy band ranging from 366 to 404 nm (Figure S14). This transition becomes essentially intraligand in character with the increase in the π-conjugation of the polypyridyl ligand, as suggested by the solvatochroism experiment, upon moving from dpq to dppz to dppn (Figure S15). The concentration-dependent UV/vis absorption spectra of **3**–**5** demonstrated that Beer’s law is obeyed at concentrations up to 20 μM (Figure S16), which suggests that the complexes are dissolved as individual molecules below this concentration. Upon excitation at the low energy band, **3**–**5** showed a very weak emission around 419, 420, and 500 nm, respectively. In contrast, **1** and **2**, which lack solvent-interacting heteroatoms, presented a high-intensity emission band with a well-defined vibrational signature (Figure S17). The nonemissive behavior of **3**–**5** in buffer is consistent with the interpretation of quenching of the excited state through vibrational deactivation processes, mediated via H-bonding.^19^

### Emission Light-Switch in the Presence of DNA

**Figure 2:**
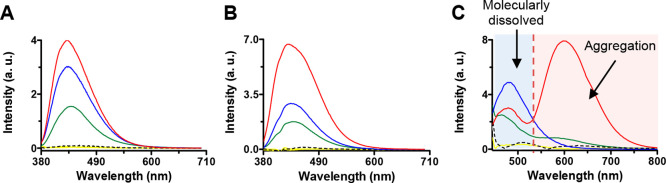
Emission spectra of complexes **3** (A), **4** (B), and **5** (C), in the absence (yellow −) or presence of different type of DNA: poly AT (red −), poly GC (blue −), G-quadruplex (green −) and CT DNA (- -). The spectra were recorded at [Pt]/[bp] ratio of 1:5 before and after 5 min of incubation with the corresponding DNA at RT. λ_ex_ = 370 nm and λ_em_ = 380–700 nm for **3**; λ_ex_ = 367 nm and λ_em_ = 377–700 for **4**; and λ_ex_ = 430 nm and λ_em_ = 445–800 nm for **5**. Note: 1% DMSO was used to ensure completely solubility of complex **5**. Tris–HCl buffer (5 mM, 50 mM NaCl, pH 7) was used in all cases, except for G-quadruplex DNA that required Tris–HCl (5 mM, 50 mM KCl, pH 5.5).

The nonemissive behavior of **3**–**5**, together with their aggregation properties in buffer, prompted us to assess their DNA sensing abilities. The interaction of **3**–**5** was evaluated at 1:5 [Pt]/[DNA bp] ratio for 10 different types of DNA (Table S2), including single strands (ss), double strands (ds), and G-quadruplex DNAs. Complexes **3**–**5** presented negligible luminescence after the addition of CT DNA, but displayed an intense luminescence when incubated with ds poly AT, with λ_max_ centered at 438, 431, and 599 nm respectively (Figure 2). Overall, the results in Figures 2 and S12 and Tables S3–S5 show a marked preference for ds poly AT over other dsDNAs (CT DNA, poly GC, poly A·poly T, and poly G·poly C), ssDNAs (poly A, poly G, poly C, and poly T) and G-quadruplex DNAs. This is manifested with a 33.7–73.9× increase in emission when bound to ds poly AT compared to the baseline luminescence of the complexes alone. It was also noted that the emission was at a higher energy (λ_max_ = 445 nm) for **5**, like **3** and **4**, alone and in the presence of the ds CT DNA, poly GC, and G-quadruplex DNA, but a lower energy emission near 600 nm was observed exclusively in the presence of the ds poly AT and poly A·poly T, and the ss poly T. This emission band is significantly red-shifted with respect to the emission of **5** alone and is comparable in energy to its excimer emission in the solid state (λ_max_ = 607 nm, Figure S19). Moreover, emission studies in organic solvents and DMSO/H_2_O mixtures demonstrate that the aggregation of **5**, and its excimeric emission in solution, can be modulated through the formation and disruption of hydrogen-bonding interactions (Figures S20 and S21). Based on previous studies,^18^^b,c^ it was hypothesized that the AT-rich DNA sequences may provide an additional hydrophobic environment that drives the self-assembly of **5** at low complex concentration. This could be caused by the electrostatic attraction between the nucleic acid molecule and the cationic complex, resulting in an increased local concentration of **5** that promote π–π and/or metal–metal interactions.^20^ The greater negative electrostatic potential of AT-base pairs in comparison with GC-base pairs^21^ explain the preferential binding of **3**–**5** toward AT-rich DNA sequences. In addition, the higher hydrophobicity of the surfaces of the minor groove walls in ds poly AT, together with its high polymorphism and flexibility,^21^ favor the self-assembly of the dppn complex **5**. Circular dichroism measurements revealed that **5** interacts with ds poly AT without modifying the conformational structure of the DNA, which is consistent with the formation of nonintercalative DNA–drug complexes (Figure S22).

The 600 nm emission is also increased in the presence of the ss poly T. The structure adopted by poly T, which is highly flexible, with very little base-stacking interactions between the thymine bases,^20,22^ would greatly reduce the DNA base-stacking interaction with the metal complex, while favoring complex self-assembly.

To further demonstrate the selectivity of the complexes for dsDNA over RNA and proteins, their emission spectra were recorded in the presence of RNA and human serum albumin (HSA) and no detectable changes were observed compared to the baseline luminescence of **3**–**5** (Figure S23).

### Theoretical Calculations

**Figure 3:**
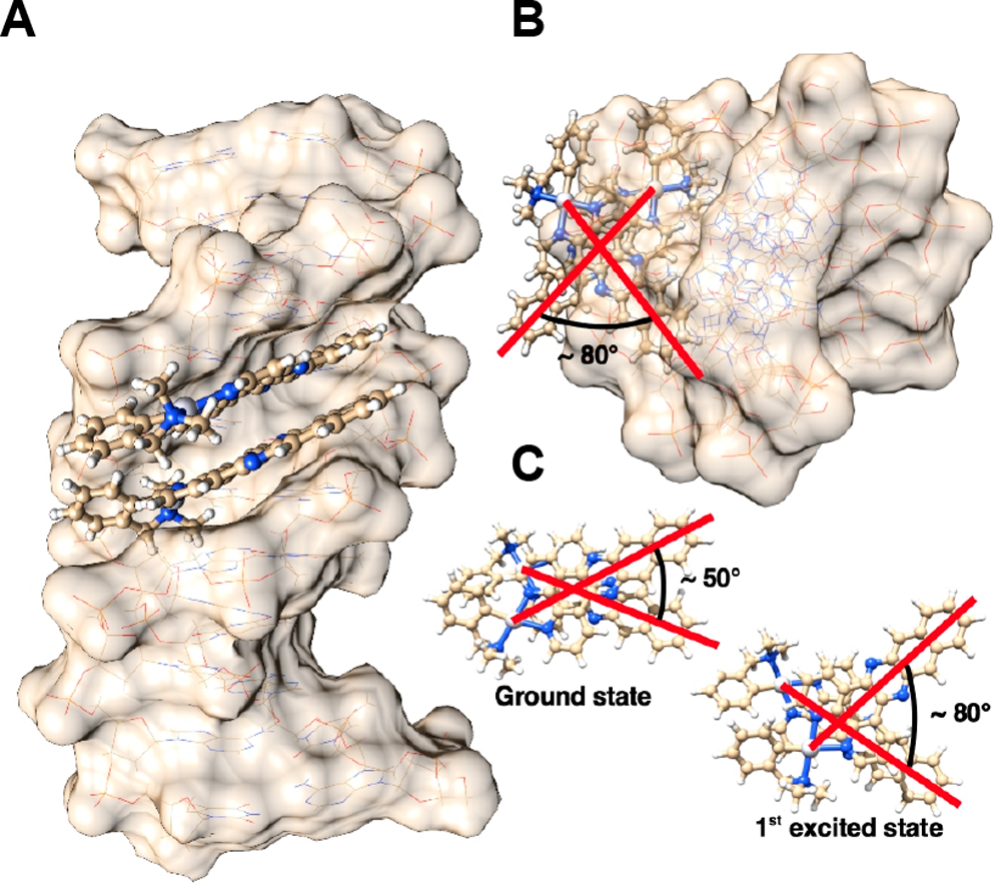
(A) Front and (B) side views of the optimized structure for the dimer of **5** after groove-binding to poly AT. (C) Angle between the dppn ligands of the isolated dimer in the ground and first excited state.

QM/MM and TD-DFT calculations were carried out in order to interpret the absorption and emission of **4** and **5** when interacting with poly AT (Figures 3 and S24–S26) and poly GC (Figures S27–S29). The calculated absorption spectrum, with **4** intercalated into the double-helical dodecanucleotide poly AT, shows the same features that were experimentally observed, specifically a slight red shift and intensity reduction for **4** in the presence of the stacked DNA bases. The calculated absorption spectrum for **5**, bound as a dimer of in the minor groove of poly AT (Figure S26), also matched well with experimental values, with an increase of intensity of the longer wavelength absorption. A similar agreement with the experimental spectra was obtained for the calculated absorption of **4** and **5** intercalated into poly GC (Figure S29).

To evaluate the emission properties of **4** and **5** metal complexes, optimization of their geometry in the first excited state was performed. The results obtained support the conclusion that both emissions come from singlet–singlet transitions, S1 → S0, as the first triplet transition occurs at a very low frequency and longer wavelength (λ_em_ = 685.24 and 1306.2 nm for **4** and **5**, respectively). The analysis of the molecular orbitals (MO) involved in the transition indicate that the dominant emission process is intraligand, due to a small contribution of the metal in the excited state.^23^ The calculated emission for the DNA intercalated complex, **4**–poly AT (λ_em_ = 417 nm; *f* = 0.0012), nicely matches with the experimental emission (λ_em_ = 431 nm). This correlation is not as close for the intercalated complex **5**–poly AT (λ_calc._= 518 nm; *f* = 0.0327 vs λ_exp._ = 599 nm). Thus, in order to determine if the emission of **5** could be explained through DNA-induced self-assembly, the emission for the dimer of **5** was also modeled. Interestingly, the excited state geometry of the stacked dimer is comparable to that observed for the dimer inserted in the minor groove (Figure 3). The angle between the aromatic ligands of the two stacked complexes is about 50° in the ground state of the isolated dimer, while it undergoes an increase of about 30° in the ground state when inserted within the minor groove of poly AT (Figure 3B). This angle is also ∼80° in the first excited state of the isolated dimer (Figure 3C). The groove-binding structure, obtained by QM/MM calculations, shows that AT-regions provide a favorable constraining environment for the dimer of **5**, which assumes a structure very near to its first excited state, from which the emission can be easily observed, compared to the isolated dimer. The first singlet transition occurs at a shorter wavelength (λ_calc._= 528 nm; *f* = 0.0252), and in our opinion the disagreement with the experimental emission is due to the neglected stabilizing contribution of the DNA on the geometric and electronic structure of the DNA-bound metal complexes. The analysis of the MO indicates that the transition can be defined as a metal-perturbed interligand transition, i.e., between the two stacked aromatic ligands (Figure S30).

### DNA Binding Mode

**Figure 4:**
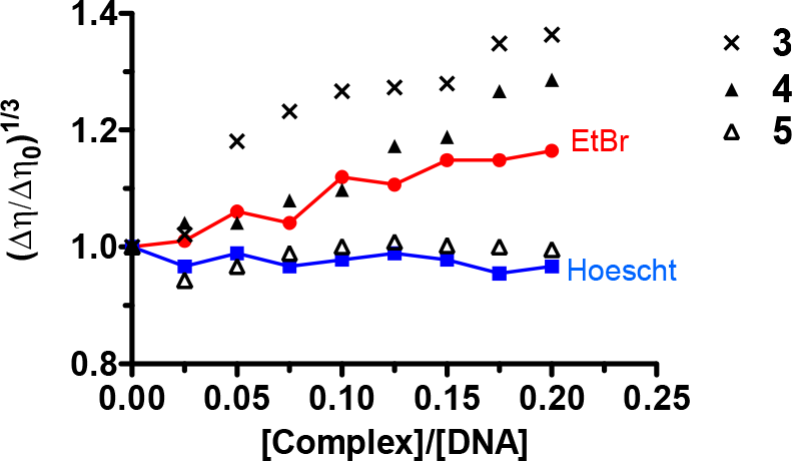
Changes in the viscosity of 200 μM CT DNA upon addition of **3** (×), **4** (▲) and **5** (△) in Tris–HCl (5 mM, 50 mM NaCl, pH 7) at RT as a function of the indicated metal complex/DNA molar ratio. EtBr (red ●) and Hoechst 33258 (blue ■) were used as controls for intercalative and minor groove binding modes, respectively.

It is interesting to note that the oligonucleotide-induced self-assembly does not occur for shorter *N,N*-diimine ligands. Presumably, the smaller π-surfaces in these metal complexes produce weaker self-associated aggregates, and as a result, **3** and **4** interact more strongly via intercalation with the nucleic acid bases. The distinct binding modes of **3**–**5** with DNA were further probed by viscometry and gel electrophoresis. Intercalation between base pairs increases the contour length of DNA, and consequently intercalators induce an increase in the viscosity of the DNA solution, with an associated enhancement in the viscosity increment, Δη, of the DNA solution. In contrast, groove binders have no effect on Δη.^24^Figure 4 shows an increased in Δη for **3** and **4** but not **5**, confirming that **5** is not an intercalator. Interestingly, the intercalation properties of **3**–**5** decrease with the increasing length of the polypyridyl ligand, which may indicate that **4** aggregates in a similar fashion to **5**. Agarose gel electrophoresis with pUC19 plasmid DNA ultimately confirmed the distinct mode of interaction of the complexes with DNA (Figure S31). Complexes **3** and **4** induced a dose-dependent effect on the DNA mobility, with decreased migration and smearing of the bands, consistent with intercalation in the plasmid DNA.^25^ Complex **4** interacts with plasmid DNA at very low concentrations (15 μM), causes significant unwinding, and provokes a loss of EtBr staining of the DNA from 125 μM. In contrast, **5** does not alter the helical winding of DNA to any significant extent at low concentrations, although a loss of EtBr signal is also observed starting at 125 μM. The reduction in the EtBr emission can be ascribed to DNA precipitation at high complex concentrations.

### Biological Activity

Given that DNA sequence specificity contributes significantly to the cytotoxic potency of several antitumor agents,^26^ the antiproliferative activity of **3**–**5** (Table 1) was evaluated and compared with the metallating agent of reference, cisplatin (CDDP), against two-dimensional A549 cells and its 3D tumor model. In contrast to two-dimensional cell culture, 3D tumor spheroids mimic the complexity of *in vivo* tumors, exhibiting the phenomena of multicellular resistance (MCR),^27^ which is manifested in diminished efficacy of chemotherapeutics, often reduced to levels similar to *in vivo* activities. Complexes **3**–**5** showed IC_50_ values in the low micromolar range in 2D, with the values of **4** and **5** being comparable to those of CDDP. However, a reduction in activity was observed when tumor spheroids of ca. 600 μm in diameter were dosed with the compounds, with the IC_50_ values for **3** and **5** reduced to 70 μM. In contrast, significant cytotoxicity was observed for **4**, with an IC_50_ value of 35 μM, compared to 34 μM for CDDP. It is promising that the MCR value for **3** and **4** is lower than that of CDDP, given that the MCR value for CDDP is much lower than for many other chemotherapeutics.^27c^

**Table 1:** Cytotoxicity IC_50_ (μM) and MCR Index at 48 h for **3**–**5** and CDDP and Platinum–DNA Binding Levels after 2 h Treatment with the Compounds

complex	A549^a^	A549 spheroid	MCR index^b^	ng Pt/μg DNA^c^
**3**	5.45 ± 0.22	74.15 ± 1.12	13.6	77.42 ± 10.02
**4**	1.20 ± 0.13	35.08 ± 1.06	29.2	178.63 ± 29.83
**5**	1.43 ± 0.04	78.69 ± 1.11	55.0	51.75 ± 5.82
**CDDP**	0.96 ± 0.19	34.18 ± 1.13	35.6	39.78 ± 6.32

a Human alveolar adenocarcinoma cell line.

b The multicellular resistance (MCR) index is the ratio of the spheroid and the monolayer culture IC_50_ values.

c Measurements indicate the Pt bound to DNA after 2 h of treatment of A549 cells with 20 μM of the compounds. The results are presented as mean ± SD of 2 independent experiments with 4 replicates each.

In order to investigate whether the antiproliferative activity of the investigated complexes might be connected with their DNA binding abilities, metal levels in nuclear DNA isolated from A549 cells were determined by inductively coupled plasma mass spectrometry (ICP-MS) after 2 h exposure to each of the compounds (20 μM; Table 1). The amount of platinum associated with the nuclear DNA was higher for the most active compound **4**, while **3** and **5** presented values on the order of that of CDDP. Interestingly, the results are in good agreement with the activity of **3**–**5** in the 3D model. It is noteworthy that these complexes, which do not form bonds to the DNA, unlike CDDP, have sufficiently high DNA binding affinities to both cause cytotoxic effects and to remain bound to the nucleic acid through the isolation procedure. However, these data cannot at this time be directly correlated with the DNA sequence specificity displayed by the complexes.

## Conclusions

In summary, amphiphilic cyclometalated Pt^II^ complexes have been shown to be capable of aggregating in an aqueous environment. The self-assembly process is reversible, and in the case of **5**, it can be selectively promoted in the presence of poly AT and poly A·poly T DNA, but not significantly with other sequences. This aggregate only forms when the π-conjugated surface at the polypyridyl ligand is large enough, as was not observed for **3** or **4**. The DNA-driven aggregation of **5** turns on excimer emission at 600 nm. The stacked structure of two complexes within the minor groove of the DNA, mediated by π–π interactions between the dppn ligands, was supported by TD-DFT and QM/MM calculations. Viscometry and gel electrophoresis indicated no intercalation interaction for **5**, while **3** and **4** were able to stack between the DNA base-pairs. All the complexes showed anticancer activity in 2D models, and **4** retained significant activity in spheroids, with a good MRC value. The work presented herein provides further insights for constructing future molecular reporters or theranostic agents based on the formation of supramolecular Pt^II^ nanostructures.

## Experimental Section

### Materials and Instrumentation

Unless otherwise noted, preparations were carried out under atmospheric conditions. Pt(DMSO)_2_Cl_2_,^28^ Pt(dmba)(DMSO)Cl^29^ and dppz-type ligand^30^ were prepared using reported procedures. All other reagents were obtained from commercial sources and used without further purification. A549 cell line was obtained from American Tissue Culture Collection (ATCC, U.S.A.).

The C, H, N, and S analyses were performed with a Carlo Erba model EA 1108 microanalyzer. The ^1^H, ^13^C, and ^195^Pt NMR spectra were recorded on a Bruker AC 300E or a Bruker AV 400 spectrometer. Chemical shifts are cited relative to SiMe_4_ (^1^H and ^13^C, external) and Na_2_[PtCl_6_] (^195^Pt, external). ESI mass analyses were performed on a HPLC/MS TOF 6220. The isotopic distribution of the heaviest set of peaks matched very closely that calculated for the formulation of the complex cation in every case. UV/vis spectroscopy was carried out on a PerkinElmer Lambda 750 S spectrometer with operating software. Fluorescence measurements were carried out with a PerkinElmer LS 55 50 Hz fluorescence spectrometer. Purity and stability analyses were carried out on an Agilent HPLC 1100 series. Chromatographic analyses were carried out on a C18 column (150 mm × 4.6 mm, 5 μm particle size; WATER SounFire). CD spectra were recorded at RT on an Applied PhotophysicsP*-180 spectrometer with a 75 W xenon lamp using a computer for spectral subtraction and smooth reduction. Viscosity and density were measured with the Anton Paar DMA 5000 M density meter, which includes the module Lovis 2000 ME rolling-ball microviscometer. Agarose gels were digitally imaged using a BioRad ChemiDoc System.

#### General Procedure for the Synthesis of Cycloplatinated Pt^II^ Complexes **1**–**4**

To a solution of [Pt(dmba)(DMSO)Cl] (2.26 mmol, 100 mg) in acetone (10 mL) was added AgNO_3_ (2.26 mmol, 38 mg). The resulting solution, protected from light, was stirred for 2 h at RT. The formed AgCl was filtered through a plug of Celite and the corresponding bidentate ligand was added in 1:1 molar ratio to the filtrate. The solution, which immediately turned to yellow, was stirred for 24 h at RT until the appearance of a yellow precipitate. The suspension was concentrated; the solid filtered and washed with a minimum volume of ether. If required, column chromatography was carried out on silica gel using CH_2_Cl_2_/MeOH (95:5) as eluent.

#### [Pt(dmba)(bpy)](NO_3_) (**1**)

Yellow solid. Yield: 51%. Anal. Calcd for **1** C_19_H_20_N_4_O_3_Pt: C, 41.68; H, 3.68; N, 10.23. Found: C, 41.48; H, 3.59; N, 10.02. ^1^H NMR (400 MHz, CDCl_3_): δ 9.30 (d, 1H, H^6′^, *J*_HH_ = 5.2 Hz,), 9.09 (d, 1H, H^6^, *J*_HH_ = 5.6 Hz, Pt satellites are observed as shoulders), 8.66 (d, 1H, H^3^ or H^3′^, *J*_HH_ = 8.0 Hz), 8.60 (d, 1H, H^3^ or H^3′^, *J*_HH_ = 8.0 Hz), 8.26 (m, 2H, H^4^+H^4′^), 8.04 (m, 1H, H^5′^), 7.57 (m, 1H, H^5^), 7.15 (m, 4H, H^dmba^), 4.19 (s, 2H, NCH_2_, Pt satellites are observed as shoulders), 3.19 (s, 6H, N(CH_3_)_2_, Pt satellites are observed as shoulders).^13^C{^1^H} NMR (75.4 MHz, CDCl_3_): δ 153.32 (*C*H^6^), 149.84 (*C*H^6′^), 140.69 (*C*H^4^ or *C*H^4′^), 140.31 (*C*H^4^ or *C*H^4′^), 134.35 (*C*H^dmba^), 129.11 (*C*H^5′^), 127.57 (*C*H^5^), 126.79 (*C*H^dmba^), 125.54 (*C*H^dmba^), 124.90 (*C*H^3^ or *C*H^3′^), 124.68 (*C*H^3^ or *C*H^3′^), 121.83 (*C*H^dmba^), 78.32 (N*C*H_2_); 53.44 (N(*C*H_3_)_2_). ^195^Pt NMR (86.28 MHz, CDCl_3_): δ −3023.78 (s). ESI-MS (pos. ion mode, H_2_O): *m*/*z* 485.1307 ([M]^+^, calcd 485.1307). UV/vis in Tris buffer, λ_max_ (ε) = 206 (42200), 246 (19100), 310 (9700), 320 (10500), 359 (3600 M^–1^ cm^–1^).

#### [Pt(dmba)(phen)](NO_3_) (**2**)

Yellow solid. Yield: 56%. Anal. Calcd for **2** C_21_H_20_N_4_O_3_Pt: C, 44.13; H, 3.53; N, 9.80. Found: C, 44.35; H, 3.16; N, 9.82. ^1^H NMR (300 MHz, CDCl_3_): δ 10.25 (d, 1H, H^9^, *J*_HH_ = 4.2 Hz), 9.49 (dd, 1H, H^2^, *J*_HH_ = 4.5 Hz, *J*_HH_ = 0.9 Hz, *J*_HPt_ = 44.8 Hz), 8.89 (dd, 1H, H^4^, *J*_HH_ = 8.1 Hz, *J*_HH_ = 0.9 Hz), 8.76 (dd, 1H, H^7^, *J*_HH_ = 8.1 Hz, *J*_HH_ = 0.9 Hz), 8.60 (m, 1H, H^8^), 8.13 (m, 2H, H^5^+H^6^), 7.95 (dd, 1H, H^3^, *J*_HH_ = 8.1 Hz, *J*_HH_ = 5.4 Hz), 7.21 (m, 4H, H^dmba^), 4.28 (s, 2H, NCH_2_, *J*_HPt_ = 44.1 Hz), 3.39 (s, 6H, N(CH_3_)_2_, *J*_HPt_ = 34.5 Hz).^13^C{^1^H} NMR (75.4 MHz, CDCl_3_): δ 152.79 (*C*H^2^+*C*H^9^), 139.03 (*C*H^4^ or *C*H^7^), 138.75 (*C*H^4^ or *C*H^7^), 133.97 (*C*H^dmba^), 128.51 (*C*H^8^), 128.77 (*C*H^5^ or *C*H^6^), 127.08 (*C*H^5^ or *C*H^6^), 126.46 (*C*H^dmba^), 125.55 (*C*H^3^), 125.30 (*C*H^dmba^), 121.80 (*C*H^dmba^), 78.21 (N*C*H_2_); 53.77 (N(*C*H_3_)_2_). ^195^Pt NMR (86.28 MHz, CDCl_3_): δ −3076.705 (s). ESI-MS (pos. ion mode, H_2_O): *m*/*z* 509.1306 ([M]^+^, calcd 509.1307). UV/vis in Tris buffer, λ_max_ (ε) = 207 (69600), 272 (29700), 371 (3800 M^–1^ cm^–1^).

#### [Pt(dmba)(dpq)](NO_3_) (**3**)

Yellow solid. Yield: 31%. Anal. Calcd for **3** C_23_H_20_N_6_O_3_Pt: C, 44.30; H, 3.23; N, 13.48. Found: C, 44.65; H, 3.07; N, 13.62. ^1^H NMR (400 MHz, CD_3_OD): δ 9.87 (dd, 1H, H^4^ or H^9^, *J*_HH_ = 8.1 Hz, *J*_HH_ = 0.9 Hz), 9.84 (dd, 1H, H^4^ or H^9^, *J*_HH_ = 8.1 Hz, *J*_HH_ = 0.9 Hz), 9.66 (dd, 1H, H^11^, *J*_HH_ = 5.2 Hz, *J*_HH_ = 1.2 Hz), 9.61 (dd, 1H, H^2^, *J*_HH_ = 5.6 Hz, *J*_HH_ = 0.8 Hz, Pt satellites are observed as shoulders), 9.23 (m, 2H, H^6^ + H^7^), 8.372 (dd, 1H, H^10^, *J*_HH_ = 8.0 Hz, *J*_HH_ = 5.2 Hz), 8.18 (dd, 1H, H^3^, *J*_HH_ = 8.0 Hz, *J*_HH_ = 5.6 Hz), 7.30–7.16 (m, 4H, H^dmba^), 4.37 (s, 2H, NCH_2_, Pt satellites are observed as shoulders), 3.26 (s, 6H, N(CH_3_)_2_). ^13^C{^1^H} NMR (75.4 MHz, CD_3_OD): δ 156.18 (*C*H^2^), 152.66 (*C*H^1^), 148.32 (*C*H^6^+*C*H^7^), 137.48 (*C*H^4^ or *C*H^9^), 137.10 (*C*H^4^ or *C*H^9^), 135.59 (*C*H^dmba^), 128.797 (*C*H^dmba^), 128.65 (*C*H^dmba^), 127.58 (*C*H^10^), 126.55 (*C*H^3^), 122.91 (*C*H^dmba^), 78.96 (N*C*H_2_), 53.76 (N(*C*H_3_)_2_). ^195^Pt NMR (86.28 MHz, CD_3_OD): δ −3084.045 (s). ESI-MS (pos. ion mode, H_2_O): *m*/*z* 561.1363 ([M]^+^, calcd 561.1368). UV/vis in Tris buffer, λ_max_ (ε) = 207 (61700), 259 (49400), 366 (3900 M^–1^ cm^–1^).

#### [Pt(dmba)(dppz)](NO_3_) (**4**)

Yellow solid. Yield: 40%. Anal. Calcd for **4** C_27_H_22_N_6_O_3_Pt: C, 48.14; H, 3.29; N, 12.48. Found: C, 48.10; H, 3.57; N, 12.71. ^1^H NMR (400 MHz, CD_3_OD): δ 9.79 (dd, 1H, H^11^, *J*_HH_= 6.0 Hz, *J*_HH_ = 1.2 Hz), 9.75 (dd, *J*_HH_= 8.4 Hz, *J*_HH_ = 1.2 Hz, 1H, H^4^), 9.51 (dd, 1H, H^13^, *J*_HH_= 5.6 Hz, *J*_HH_ = 1.6 Hz), 9.47 (dd, 1H, H^2^, *J*_HH_ = 5.6 Hz, *J*_HH_ = 1.6 Hz, Pt satellites are observed as shoulders), 8.27 (m, 3H, H^7^ + H^8^ + H^12^), 8.06 (dd, 1H, H^3^, *J*_HH_ = 8.4 Hz, *J*_HH_ = 4.5 Hz), 8.01 (m, 2H, H^6^ + H^9^), 7.22–7.09 (m, 4H, H^dmba^), 4.28 (s, 2H, NCH_2_, Pt satellites are observed as shoulders), 3.16 (s, 6H, N(CH_3_)_2_, Pt satellites are observed as shoulders). ^13^C{^1^H} NMR (75.4 MHz, CD_3_OD): δ 156.17 (*C*H^2^), 152.58 (*C*H^13^), 137.65 (*C*H^11^), 137.22 (*C*H^4^), 135.62 (*C*H^dmba^), 133.92 (*C*H^6^ + *C*H^9^), 130.93 (*C*H^7^+*C*H^8^), 129.09 (*C*H^12^), 128.93 (*C*H^3^), 127.63 (*C*H^dmba^), 126.61 (*C*H^dmba^), 122.95 (*C*H^dmba^), 78.98 (N*C*H_2_), 53.79 (N(*C*H_3_)_2_).^195^Pt NMR (86.28 MHz, CD_3_OD): δ −3079.527 (s). ESI-MS (pos. ion mode, H_2_O): *m*/*z* 611.1522 ([M]^+^, calcd 611.1525). UV/vis in Tris buffer, λ_max_ (ε) = 207 (48600), 278 (40600), 367 (8700), 382 (8500 M^–1^ cm^–1^).

#### Procedure for the Synthesis of [Pt(dmba)(dppn)](NO_3_) (**5**)

To a solution of [Pt(dmba)(DMSO)Cl] (2.26 mmol, 100 mg) in acetone (10 mL) was added AgNO_3_ (2.26 mmol, 38 mg). The resulting solution was stirred for 2 h at RT, isolated from light. The formed AgCl was filtered through a plug of Celite and the filtrate was concentrated to dryness. The residue was redissolved in EtOH (10 mL), and dppn was added in 1:1 molar ratio. The resulting suspension was stirred 24 h at reflux (78 °C). The orange precipitate was filtered under vacuum and washed with EtOH and cold hexane. The solid was further purified by dissolving the impurities in hot CHCl_3_ (10 mL).

Orange solid. Yield: 68%. Anal. Calcd for **5** C_31_H_24_N_6_O_3_Pt: C, 51.45; H, 3.34; N, 11.61. Found: C, 51.66; H, 3.55; N, 11.69. ^1^H NMR (>8 × 10^–3^ M, 400 MHz, DMSO-*d*_6_): δ 9.78 (m, 2H), 9.62 (m, 1H), 9.47 (m, 1H, Pt satellites are observed as shoulders), 8.96 (broad s, 2H), 8.39 (m, 1H), 8.28 (m, 3H), 7.73 (m, 2H), 7.30–7.17 (m, 4H, H^dmba^), 2.13 (s, 2H, NCH_2_), 3.16 (s, 6H, N(CH_3_)_2_). ^13^C{^1^H} NMR (100.5 MHz, DMSO-*d*_6_): δ 155.83, 152.58, 151.07, 149.39, 149.05, 140.46, 140.02, 138.32, 136.89, 136.58, 135.48, 135.17, 130.72, 130.42, 129.35, 128.70, 127.45, 126.20, 122.93, 76.97, 52.80 ppm.^195^Pt NMR (86.28 MHz, DMSO-*d*_6_): δ −3057.567 (s). ESI-MS (pos. ion mode, H_2_O 4% DMSO): *m*/*z* 661.1687 ([M]^+^, calcd 661.1682). UV/vis in Tris buffer (2% DMSO), λ_max_ (ε) = 242 (31200), 268 (3800), 313 (40200), 404 (10100 M^–1^ cm^–1^).

### Crystal Analyses

Single crystals suitable for X–ray diffraction analysis were obtained from the slow diffusion of hexane into a saturated solution of **4** in CH_2_Cl_2_/hexane and from the slow evaporation of a D_2_O solution of **2**. A summary of crystal data collection and refinement parameters for all compounds are given in Tables S6 and S9 in Sections S6 and S7, respectively. Crystals were mounted on glass fibers and transferred to the cold gas stream of the diffractometer Bruker Smart APEX. Data were recorded with Mo Kα radiation (λ = 0.71073 Å) in ω scan mode. Absorption correction for the compound was based on multiscans.

Both structures were solved by direct methods (SHELXS-97);^31^ refinement was done by full-matrix least-squares on *F*^2^ using the SHELXL-97 program suite,^31^ with empirical (multiscan) absorption correction with SADABS (Bruker).^32^ All esds (except the esd in the dihedral angle between two l.s. planes) are estimated using the full covariance matrix. The cell esds are taken into account individually in the estimation of esds in distances, angles and torsion angles; correlations between esds in cell parameters are only used when they are defined by crystal symmetry. An approximate (isotropic) treatment of cell esds is used for estimating esds involving l.s. planes. All non-hydrogen positions were refined with anisotropic temperature factors. Hydrogen atoms for aromatic CH, aliphatic CH, CH_2_ and methyl groups were positioned geometrically (C–H = 0.95 Å for aromatic CH, C–H = 1.00 Å for aliphatic CH, C–H = 0.99 Å for CH_2_, and C–H = 0.98 Å for CH_3_) and refined using a riding model (AFIX 43 for aromatic CH, AFIX 23 for CH_2_, and AFIX 137 for rotating group for CH_3_), with *U*_iso_(H) = 1.2*U*_eq_(CH) and *U*_iso_(H) = 1.5*U*_eq_(CH_3_). Graphics were drawn with DIAMOND (Version 3.2).^33^ Analyses on the supramolecular C–H···O, C–H···π and π–π stacking interactions were done with PLATON for Windows.^34^ The crystallographic data have been deposited with the Cambridge Crystallographic Data Center (CCDC-number 1515373 for **4** and CCDC-number 1998467 for **2**). These data can be obtained free of charge via www.ccdc.cam.ac.uk/data_request/cif.

A disordered nitrate anion (besides the ordered one), a disordered hexane molecule, and two disordered (partly occupied) CH_2_Cl_2_ molecules could be located before SQUEEZE for the two symmetry-independent Pt molecules. The electron count is hexane = 48, NO_3_ = 31, and CH_2_Cl_2_ = 48. If there are 2 hexane molecules per unit cell = 96 electrons, 2 disordered nitrate anions per unit cell = 62 electrons, and 1.5 disordered CH_2_Cl_2_ molecules per unit cell = 144 electrons, then these add up to 302 electrons per void in the unit cell. Platon^33^ had calculated/squeezed a void electron count of 282 electrons in a void volume of 932 Å^3^ per unit cell volume of 2946.1 Å^3^ [32%].

### RP-HPLC Purity and Stability Analyses

The purity of each Pt^II^ complex was analyzed using mobile phases of 0.1% formic acid in dH_2_O and 0.1% formic acid in HPLC grade CH_3_CN. Samples of Pt^II^ complex **1**–**4** were prepared in dH_2_O while complex **5** was prepared in a mixture of DMSO:dH_2_O (4:96).

Stability of the complexes was studied by checking aqueous solution (4 mM and 100 mM Cl^–^ ions) of **2** and **5** after 24 h by ^1^H NMR and HPLC. Stability of complex **5** was also studied in RPMI culture medium by HPLC. Note: Solutions of **5** always contains 4% DMSO. The gradient used is shown in Table S1.

### Solubility of **5** in dH_2_O

A saturated solution of **5** in dH_2_O was prepared and stirred for 48 h. The remaining insoluble complex was filtered off and the UV/vis spectrum of the filtrate was registered. The solubility was obtained using the calculated extinction coefficient for **5**, ε(404) = 10100 M^–1^ cm^–1^. The solubility of **5** in Tris HCl (5 mM, 50 mM NaCl, pH 7) was calculated similarly obtaining a concentration of *S* = 4.8 μM. Therefore, to avoid precipitation of the complex in the buffer medium, a minimum of 1% DMSO was always used for all experiments unless otherwise specified.

### DNA Saturation Binding

The emission of **3**–**5** was tested at 10 μM in the presence of 50 μM of each DNA given in Table S2 in a 500 μL quartz cuvette. For DNA sequences the concentration is measured in [bp] and the ratio of [Pt]/[bp] was 1:5. A final volume of 300 μL was used for all samples. Data collection was taken before and after 5 min of incubation with DNA and performed with λ_ex_ = 370 nm and λ_em_ = 380–700 nm for **3**; λ_ex_ = 367 nm and λ_em_ = 377–700 for **4**; and λ_ex_ = 430 nm and λ_em_ = 445–800 nm for **5**. Note: 1% DMSO was used to ensure completely dissolution of complex **5**.

### Circular Dichroism

The CD spectra of poly AT (50 μM) were recorded before and after 5 min of incubation at 298 K with complex **5** (10 μM). The ratio of [Pt]/[bp] was 1:5. As a blank, a solution of Tris–HCl buffer (5 mM, 50 mM NaCl, pH 7.0) and complex **5** in Tris–HCl buffer were used, respectively. Each sample was scanned twice in a range of wavelengths between 200 and 340 nm. The drawn CD spectra are the mean of two independent scans. The ellipticity values are given in millidegrees (mdeg). Note: 1% DMSO was used to ensure completely dissolution of complex **5**.

### Interaction HSA and RNA

The emission of **3**–**5** in Tris–HCl (5 mM, 50 mM NaCl, pH 7) buffer was tested at 10 μM in the presence of 50 μM of HSA (albumin human 96%, lyophilized powder, Alfa Aesar) or RNA (RNA from yeast, Roche Diagnostics GmbH) after 5 min of incubation in a 500 μL quartz cuvette. Positives controls were also measured to ensure the integrity of both HSA and RNA. For RNA, EtBr dissolved in Tris–HCl (5 mM, 50 mM NaCl, pH 7) buffer was used as control. The spectrum of EtBr (10 μM) was collected in the absence and presence of RNA (50 μM) using λ_ex_ = 450 nm, λ_em_ = 470–880 nm. The emission of the Trp residues in HSA was used as a positive control. Data were collected at λ_ex_ = 295 nm and λ_em_ = 300–550 nm.

### Viscosity Measurements

CT DNA was dissolved in aqueous Tris buffer and broken into an average of 800–1000 bp by sonication (12.5 min). Then, additions of the ligand to ≈200 μM/bp DNA were made to prepare solution with ratios [complex]/[DNA] between 0 and 0.20. The viscosity of each solution was measured at 25 °C. Results from these measurements are handled in the form of viscosity increments, Δη = η – η_0_, where η is the solution viscosity and η_0_ is that of the aqueous buffer measured in the same experimental conditions. In dilute solution, η is just slightly higher than η_0_; therefore, viscosity measurements necessarily had a precision better then 0.5% so that Δη could be determined with sufficient accuracy.

### DNA Gel Electrophoresis

Compounds were mixed with 40 μg/mL pUC19 plasmid DNA in 10 mM potassium phosphate buffer, pH 7.4. Samples were then incubated for 12 h at RT. Single and double-strand DNA break controls were prepared, and the DNA samples were resolved on agarose gels, as described previously.^35^ In brief, samples were resolved on a 1% agarose gels prepared in tris-acetate buffer with 0.3 μg of plasmid/lane. The gels were stained with 0.5 μg/mL EtBr in tris-acetate buffer at RT for 40 min, destained with tris-acetate buffer, and imaged on a ChemiDoc MP System (Bio-Rad).

### Cytotoxicity Assays

Human alveolar adenocarcinoma A549 cells were maintained in DMEM, supplemented with 10% FBS and 50 U/mL pen-strep at 37 °C with 5% CO_2_. A549 cells were assayed in Opti-MEM supplemented with 1% serum supreme and 50 U/mL pen-strep and seeded into 96 well plates at a density of 1.5 × 10^3^ cells/well followed by a 6 h incubation at 37 °C, 5% CO_2_. Cells were then dosed with serial dilutions of compound and incubated for 48 h. Cell viability was determined by measuring the conversion of resazurin to resorufin^35^ using a SpectraFluor Plus Plate Reader (Tecan). Data were fit to an equation for a sigmoidal dose response using the equation below, where y_i_ and y_f_ are the initial and final signal intensities.
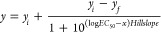


For comparison purposes, the cytotoxicity of CDDP was evaluated under the same experimental conditions. All compounds were tested in three independent studies with quadruplicate points. The compounds were dissolved in water, except **5**, which was dissolved in DMSO and diluted in the culture medium so that the final % DMSO was 0.4.

### Cytotoxicity Test on MCTSs

A549 MCTSs (diameter 600 μm) were treated by carefully replacing 50% of the medium with drug-supplemented standard medium by using an eight-channel pipet. In parallel, 50% of the solvent-containing medium was replaced by solvent-free medium for the untreated MCTSs. Three MCTSs were treated per condition and drug concentration, and the DMSO volume was less than 1% (v/v). The MCTSs were then allowed to incubate for another 48 h. The cytotoxicity of the platinum complexes toward the MCTSs was measured by the adenosine triphosphate (ATP) concentration with the Cell TiterGlo kit (Promega). After 30 min of incubation, the MCTSs were carefully transferred into black-sided, flat-bottomed 96-well plates (Corning) and mixed with a pipet for luminescence measurements on a SpectraFluor Plus Plate Reader (TECAN). Data were fit to an equation for sigmoidal dose response shown above using the Prism software package.

### Quantification of Platinum Bound to DNA

A549 cells were seeded in T25 cm^2^ flasks at high density and allowed to reach 80% confluence over 48 h. Cells were then treated with 20 μM of the tested compounds or CDDP for 2 h. Genomic DNA was isolated using DNAzol reagent (MRC) following manufacturer instructions. Extracted DNA was quantified using NanoDrop-1000 prior DNA digestion with Suprapur nitric acid 30% for 24 h. The amount of metal element platinum was determined using inductively coupled plasma mass spectrometry (ICP-MS) in Agilent ICP-MS 7900 equipment. Data were expressed as nanograms of metal per picograms of DNA. Two independent experiments were performed with *n* = 2 per sample (*n* = 4 biological independent replicates).

### Computational Details

The coordinates of an alternating deoxydodecanucleotide double helix, d(ATATATATATAT)_2_, for both intercalation and groove binding modes, were built as recently reported.^36^

The geometry of metal complex-d(ATATATATATAT)_2_ systems was fully optimized by two-layer QM/MM calculations, as implemented in the ONIOM method^37^ with the aim to perform a higher-level calculation on the intercalation pocket (high layer) and to take account of the constraining effects of the double-helical structure at lower levels of theory. In the intercalation complexes, the high layer of the model includes the sixth and seventh base pairs of the DNA models and the intercalated metal complex. In the groove binding mode, only the metal complex dimer **5** was included in the higher layer. To model the host–guest noncovalent interactions, the M06-2X^38^ DFT functional was used in the high QM layer, together with the Lanl2dz pseudopotential basis set^39^ for Pt and the dzvp basis set^40^ for the other atoms. The Amber99^41^ force field was used in the low MM layer of the QM/MM calculations, as recently described.^37^ Vibration frequency calculations, within the harmonic approximation, were performed to confirm that the two optimized geometries represented a minimum in the potential energy surface. Solvent effects were evaluated by DFT single point calculations on the high layer model extracted from the QM/MM optimized geometry, with the implicit water solvent reproduced by the polarizable continuum model (PCM).^42^ Time dependent (TD)^43^ DFT calculations were performed on the metal complexes **4** and **5** and on the stacked dimer of **5**, with the aim of optimizing the geometry of the first singlet excited state. TD-DFT calculations also were performed on the high layers of the optimized geometries, using the same M06-2X functional and basis set described above, in the presence of the implicit solvent. These allowed us to evaluate the lowest-energy singlet and triplet electronic transitions. All calculations were performed by the Gaussian 09 program package.^44^

## Supporting Information Available

HPLC method, DNA sequences tested, NMR spectra, HPLC chromatograms, absorption and emission spectra of **1**−**5**; luminescence data, CD spectra and QM/MM structures of the DNA-complexes when interacting with DNA, molecular orbitals for the dimer of **5**; agarose gels and crystallographic information (PDF)

## Accession Codes

CCDC 1515373 and 1998467 contain the supplementary crystallographic data for this paper. These data can be obtained free of charge via www.ccdc.cam.ac.uk/data_request/cif, or by emailing data_request@ccdc.cam.ac.uk, or by contacting The Cambridge Crystallographic Data Centre, 12 Union Road, Cambridge CB2 1EZ, UK; fax: +44 1223 336033.
