# Supplementary material for: Organoplatinum(II) Complexes Self-Assemble and Recognize AT-Rich Duplex DNA Sequences
Source: Inorg Chem. 2021 Jan 27;60(4):2178–87. doi: 10.1021/acs.inorgchem.0c02648 (PMC8456496; doi:10.1021/acs.inorgchem.0c02648)
Supplement: Supplementary file 1 — ic0c02648_si_001.pdf [file ic0c02648_si_001.pdf]

Supporting Information

"Organoplatinum(II) complexes self-assemble and recognize AT rich duplex DNA sequences"

Ana Zamora,[a],†,* Erin Wachter,[b] Maria Vera,[a] David Heidary, [b] Venancio Rodríguez,[a] Enrique Ortega,[a] Vanesa Fernández-Espín,[c] Christoph Janiak,[d] Edith C. Glazer,[b] Giampaolo Barone,[e],* and José Ruiz[a],*

[a] Departamento de Química Inorgánica, Universidad de Murcia, and IMIB-Arrixaca, E-30071 Murcia (Spain), Email: jruiz@um.es. and anamaria.zamora1@um.es.

[b] Department of Chemistry, University of Kentucky 505 Rose Street, Lexington, KY 40506 (USA)

[c] Departamento de Química Física, Universidad de Murcia, E-30071 Murcia (Spain)

[d] Institut für Anorganische Chemie und Strukturchemie, Heinrich-Heine-Universität Düsseldorf, D-40204 Düsseldorf (Germany)

[e] Dipartimento di Scienze e Tecnologie Biologiche, Chimiche e Farmaceutiche (STEBICEF), Università di Palermo, 90128 Palermo (Italy), Email: giampaolo.barone@unipa.it.

†Current Affiliation: Department of Chemistry, Molecular Imaging and Photonics, KU Leuven (Belgium). Email: anamaria.zamoramartinez@kuleuven.be

Table of Contents

1. Additional Tables and Figures

2. Crystal Data for **4**

3. Crystal Data for **2**

4. References

1. Additional Tables and Figures:

Table S1. HPLC method.

Time (min)	0.1% formic acid in dH_2O	0.1% formic acid in CH_3CN
0	90	10
14	10	90
18	10	90
18.1	90	10
25	90	10

Table S2. DNA sequences tested.

DNA	Buffer	Annealing Temperature /Time	5'-3' fwd	5'-3' rev
CT DNA	50 mM NaCl, 5 mM Tris, pH 7.0	10	-	-
poly GC	50 mM NaCl, 5 mM Tris, pH 7.0	90 °C/ 10 min	GCG CGC GCG CGC GCG CGC GC	GCG CGC GCG CGC GCG CGC GC
poly AT	50 mM NaCl, 5 mM Tris, pH 7.0	90 °C/ 10 min	ATA TAT ATA TAT ATA TAT AT	ATA TAT ATA TAT ATA TAT AT
poly A	50 mM NaCl, 5 mM Tris, pH 7.0	-	AAA AAA AAA AAA AAA AAA AAA	-
poly T	50 mM NaCl, 5 mM Tris, pH 7.0	-	TTT TTT TTT TTT TTT TTT TTT	-
poly G	50 mM NaCl, 5 mM Tris, pH 7.0	-	GGG GGG GGG GGG GGG GGG GGG	-
poly C	50 mM NaCl, 5 mM Tris, pH 7.0	-	CCC CCC CCC CCC CCC CCC CC CCC	-
poly A poly T	50 mM NaCl, 5 mM Tris, pH 7.0	90 °C/ 10 min	AAA AAA AAA AAA AAA AAA AAA	TTT TTT TTT TTT TTT TTT TTT
poly G poly C	50 mM NaCl, 5 mM Tris, pH 7.0	90 °C/ 10 min	GGG GGG GGG GGG GGG GGG GGG	CCC CCC CCC CCC CCC CCC CC CCC
inter G-quad	50 mM KCl, 5 mM Tris, pH 5.5	90 °C/ 5 min	TAG GGT TA	-
GC rich M	50 mM NaCl, 5 mM Tris, pH 7.0	90 °C/ 10 min	CGG CGG AAA TTA CCG CCG	CGG CGG TAA TTT CCG CCG
GC rich MM	50 mM NaCl, 5 mM Tris, pH 7.0	90 °C/ 10 min	CGG CGG AAA TTA CCG CCG	CGG CGG AAA TTA CCG CCG
AA M	50 mM NaCl, 5 mM Tris, pH 7.0	90 °C/ 10 min	CCT CGG AAA TTA CGC TTC TTC	GAA AA CGG TAA TTT CCG AGG
AA MM	50 mM NaCl, 5 mM Tris, pH 7.0	90 °C/ 10 min	CCT CGG AAA TTA CGC TTC TTC	GAA GAA CGG AAA TTA CCG AGG

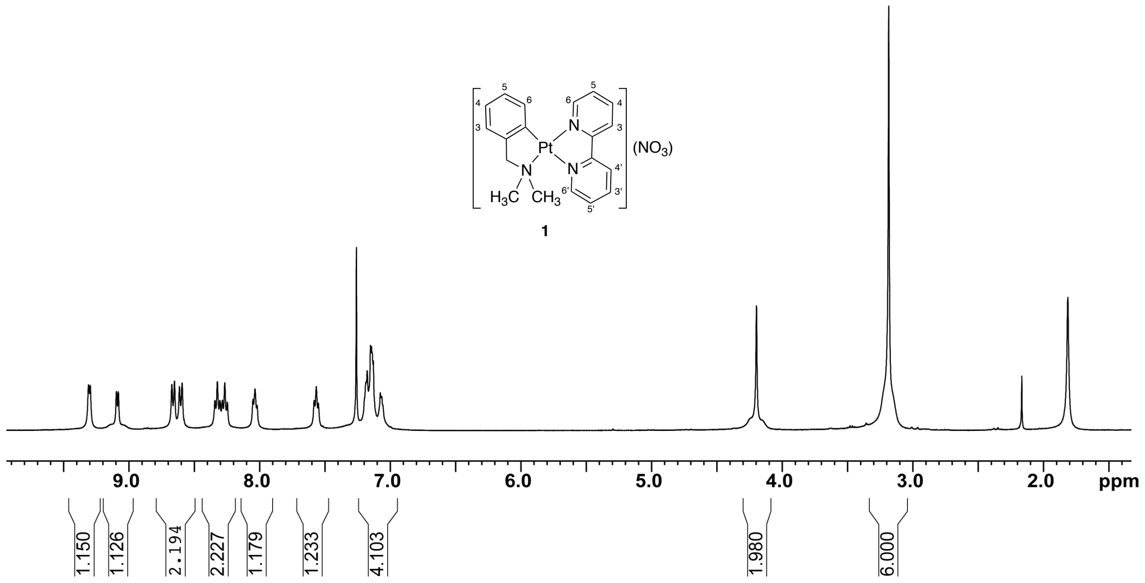

Figure S1. ^{1}H NMR spectrum of **1** (400 MHz, $CDCl_3$).

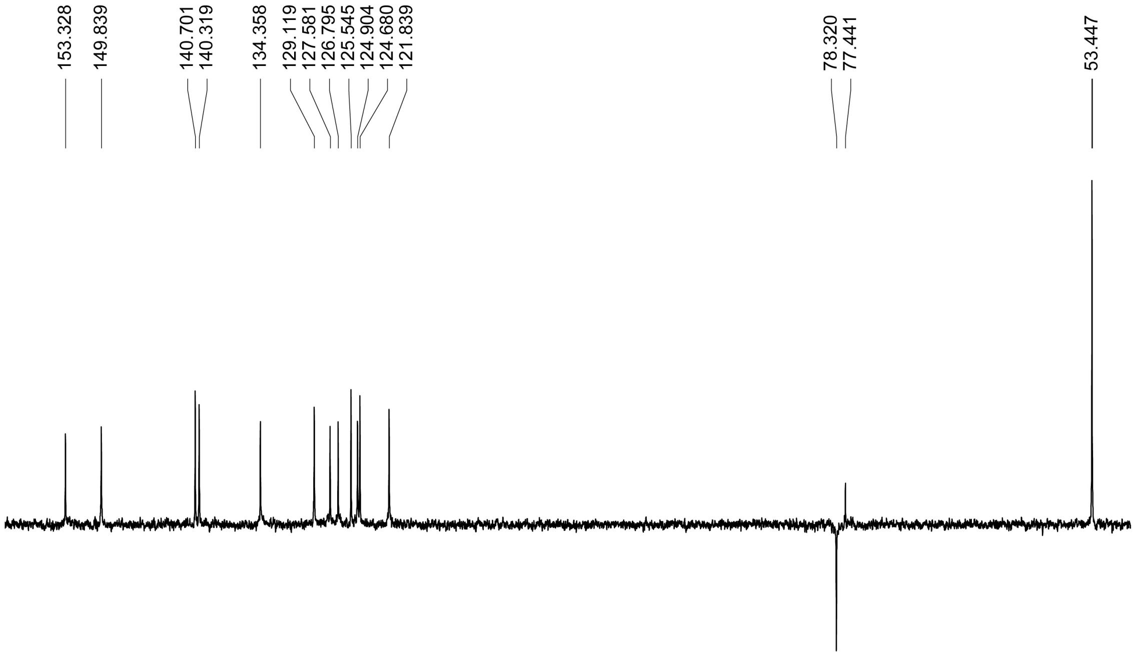

Figure S2. DEPT-135 NMR spectrum of **1** (75.4 MHz, $CDCl_3$).

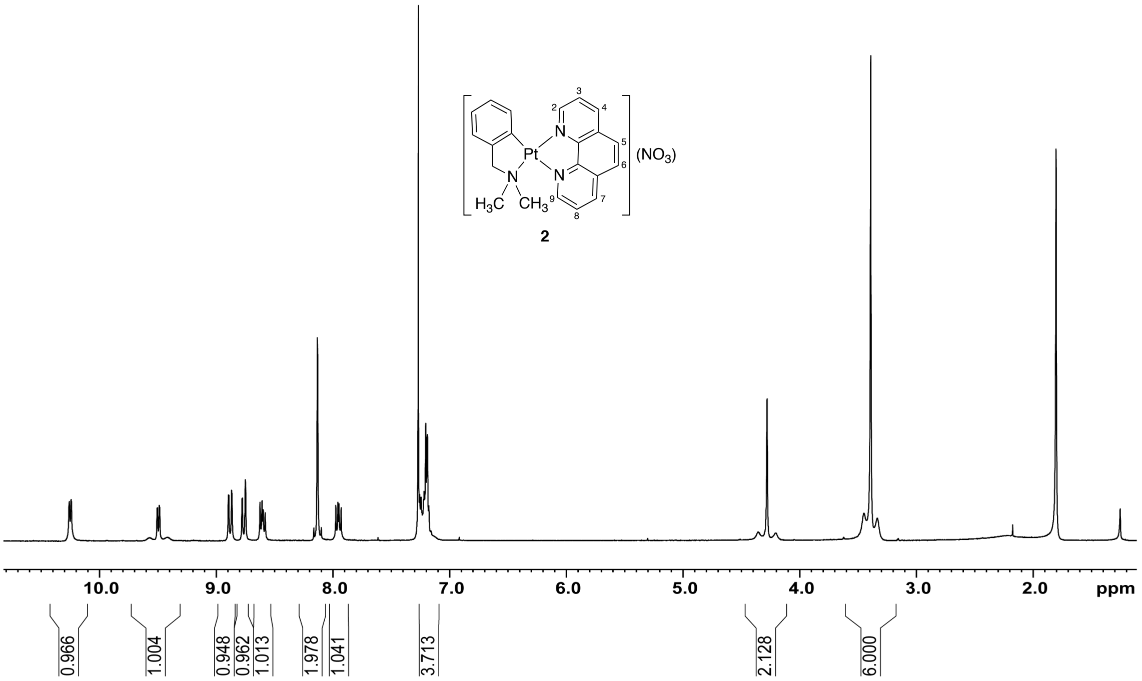

Figure S3. ^{1}H NMR spectrum of **2** (300 MHz, $CDCl_3$).

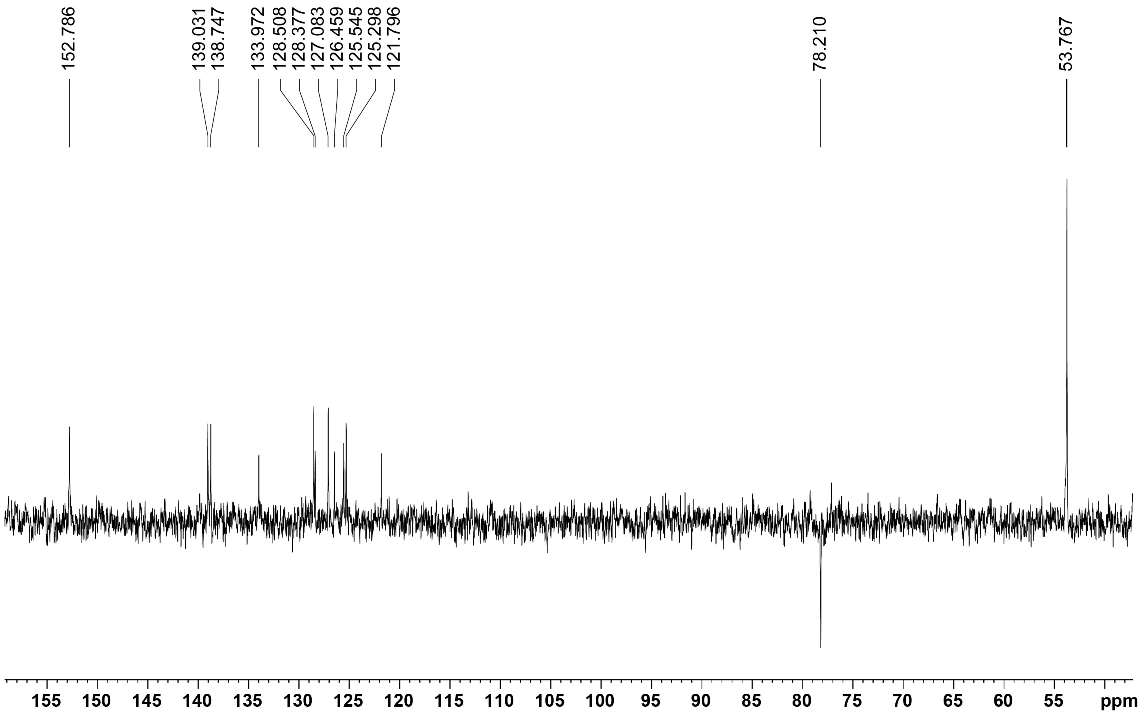

Figure S4. DEPT-135 NMR spectrum of **2** (75.4 MHz, $CDCl_3$).

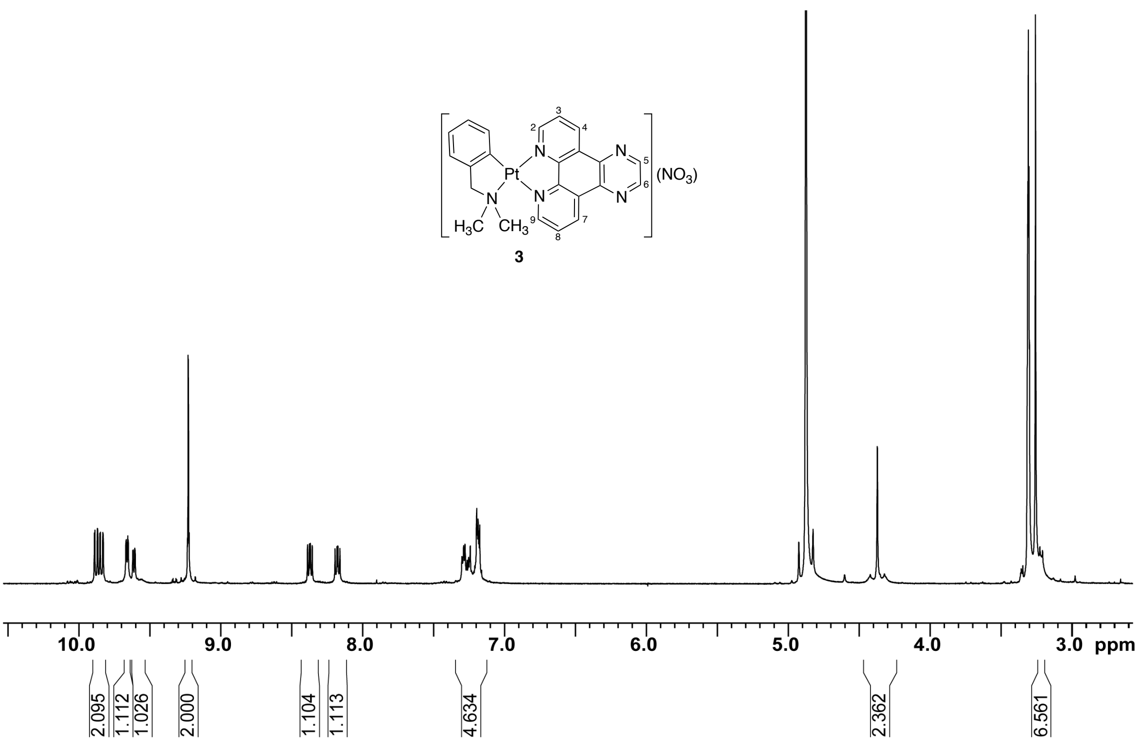

Figure S5. ^1H NMR spectrum of **3** (400 MHz, CD_3OD).

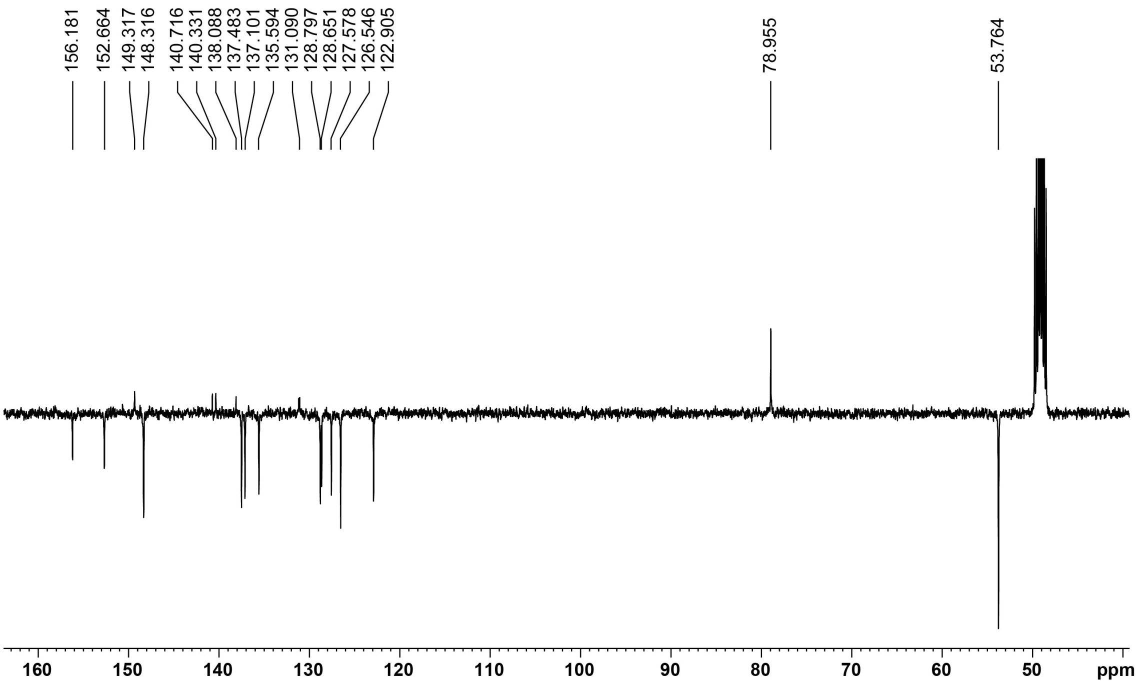

Figure S6. APT NMR spectrum of **3** (75.4 MHz, CD_3OD).

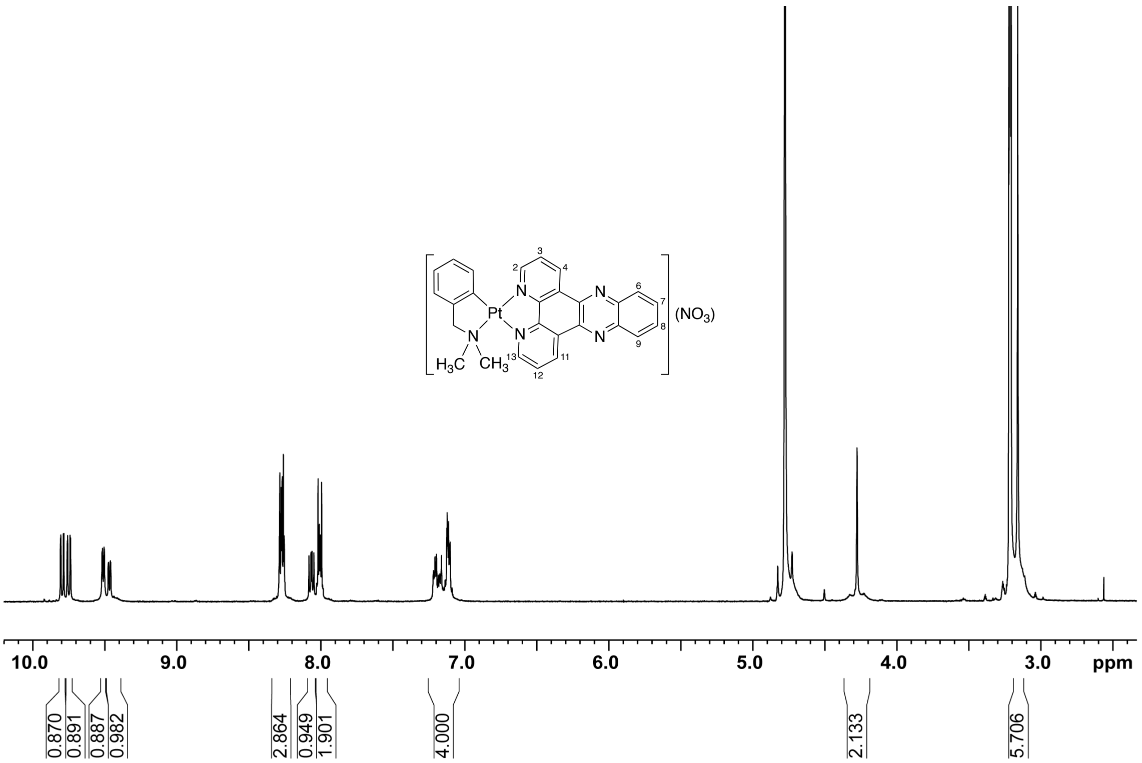

Figure S7. ^{1}H NMR spectrum of **4** (400 MHz, CD_3OD).

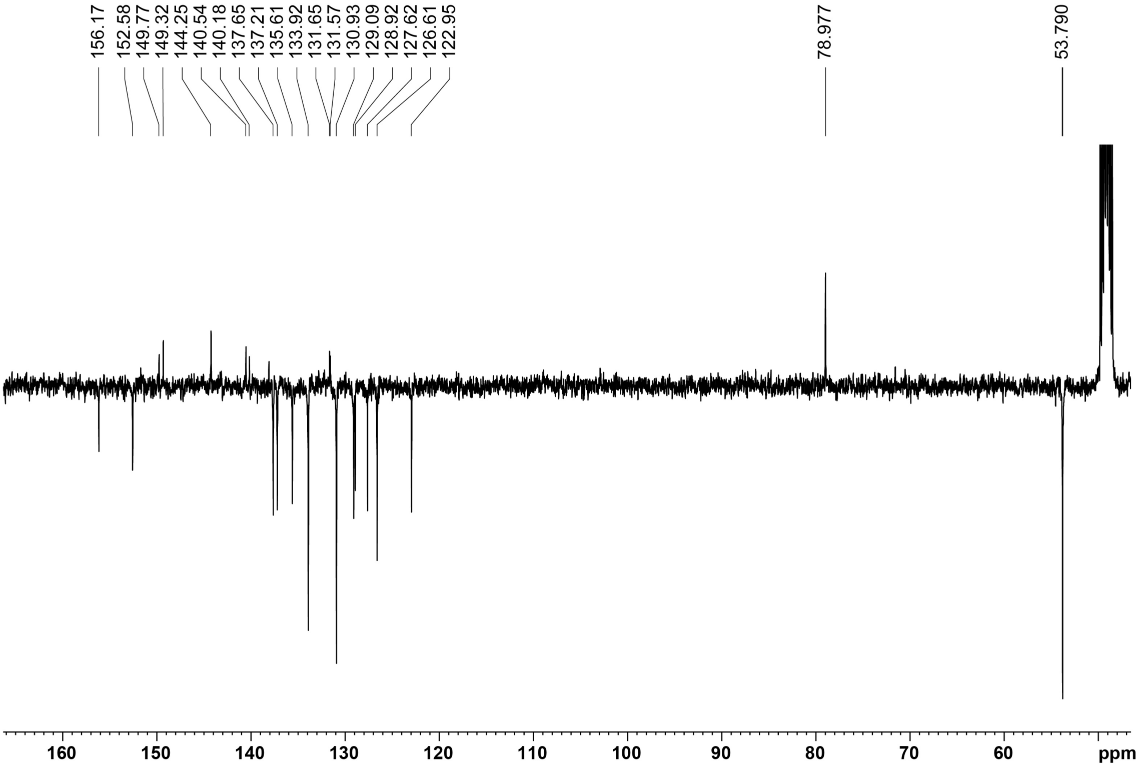

Figure S8. APT NMR spectrum of **4** (75.4 MHz, CD_3OD).

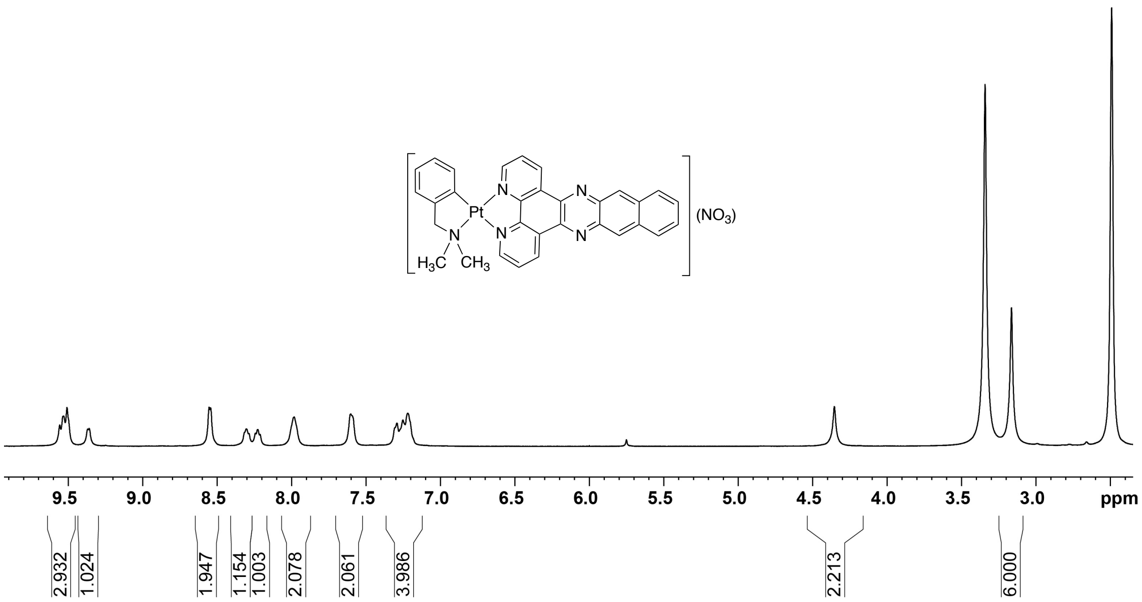

Figure S9. ^{1}H NMR spectrum of **5** (> 8 x 10^{-3} M, 400 MHz, DMSO-d_6).

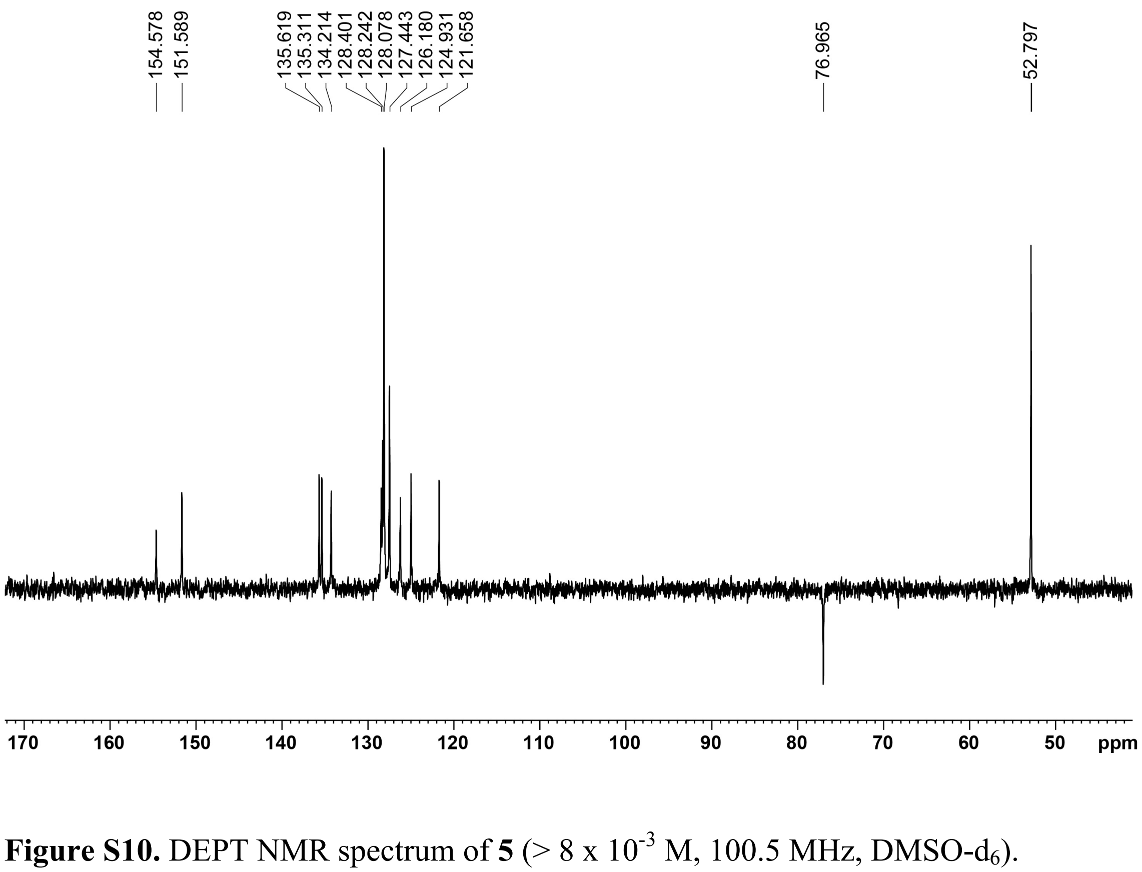

Figure S10. DEPT NMR spectrum of **5** (> 8 x 10^{-3} M, 100.5 MHz, DMSO-d_6).

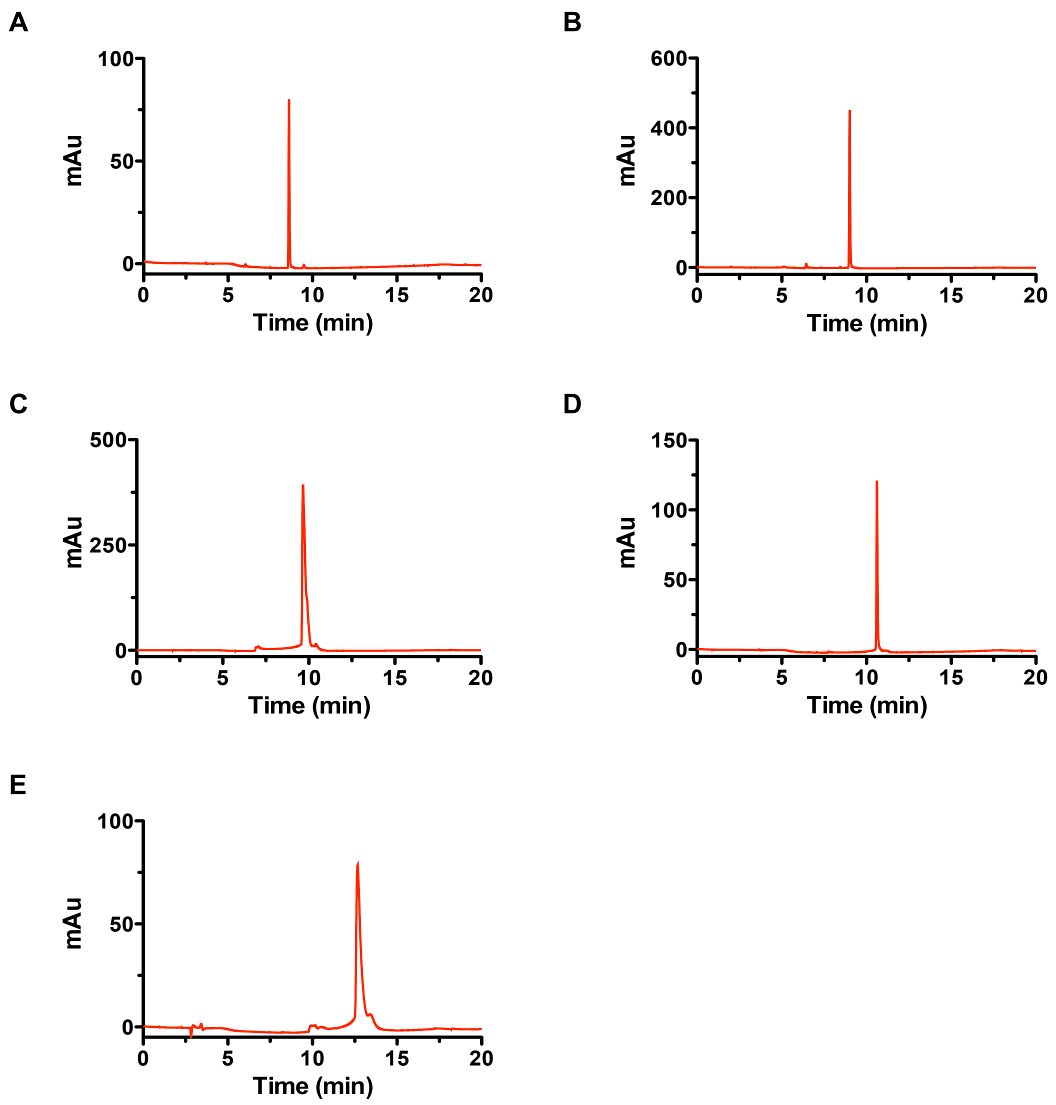

Figure S11. HPLC chromatograms of complexes in water: **1** (A), **2** (B), **3** (C), **4** (D) and **5** (E). Complex **5** was dissolved in 4% DMSO to ensure solubility.

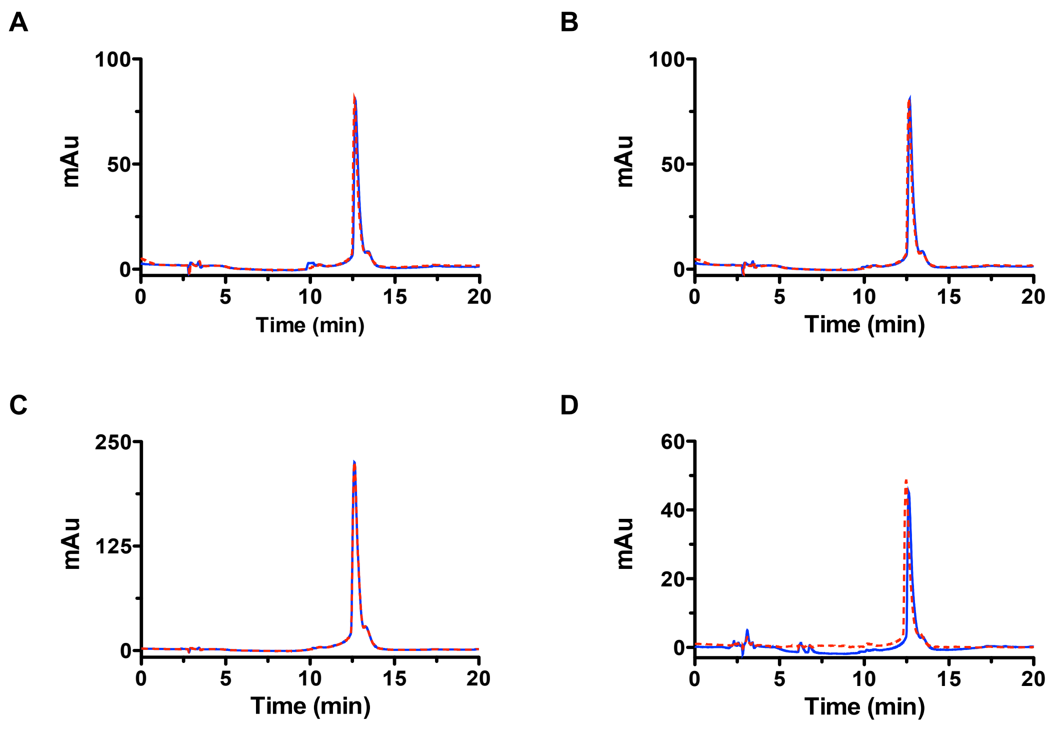

Figure S12. HPLC chromatograms of **5** (ca. 100 µM) in H_2O (A), H_2O (4 mM Cl^-) (B), H_2O (100 mM Cl^-) (C) and RPMI culture medium (D) at t= 0 h (—) and t= 24 h (– –) incubation. Each sample contain 4% DMSO.

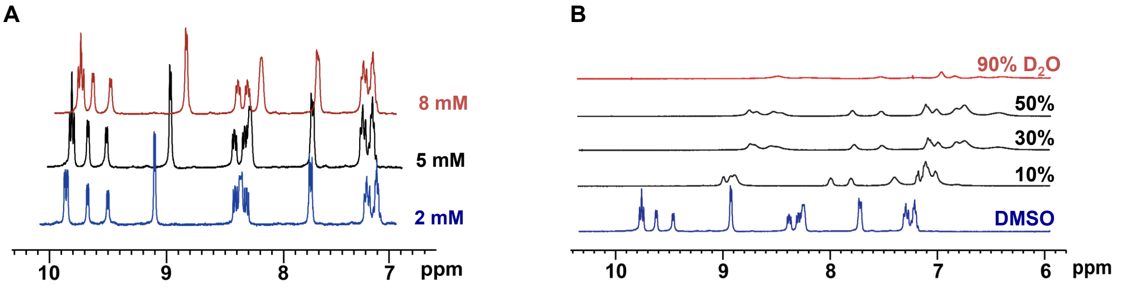

Figure S13. ^{1}H NMR spectral traces of **5** at different concentrations (A) and at different compositions of DMSO:H_2O (B). The upfield shift of the proton signals when increasing concentration together with the fact that the signals become poorly resolved as the percentage of D_2O increases are an indication of aggregate formation.

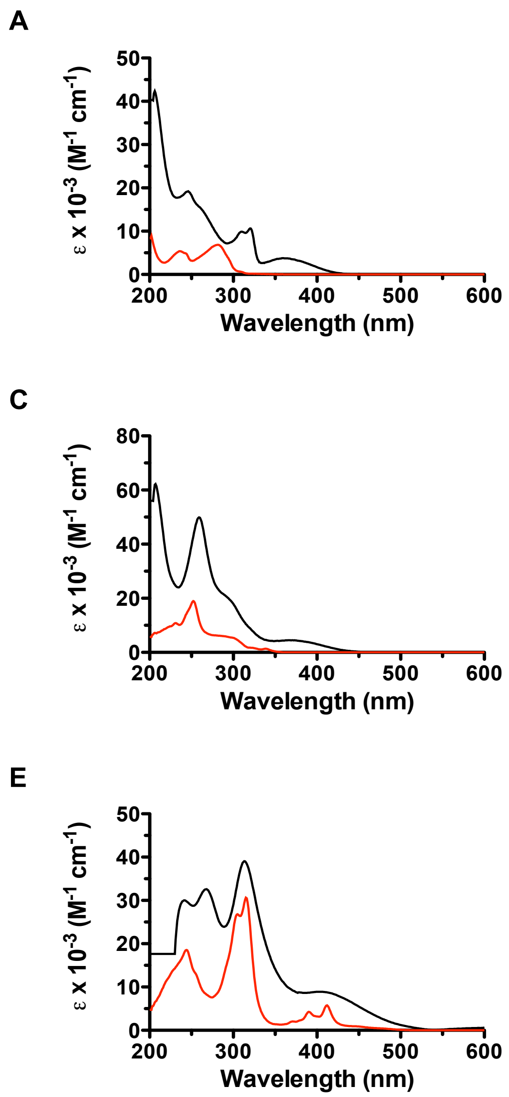

Figure S14. UV/Vis absorption spectra comparison of complexes (—, Tris buffer) and ligands (—, EtOH): **1** and bpy (A), **2** and phen (B), **3** and dpq (C), **4** and dppz (D), **5** and dppn (E).

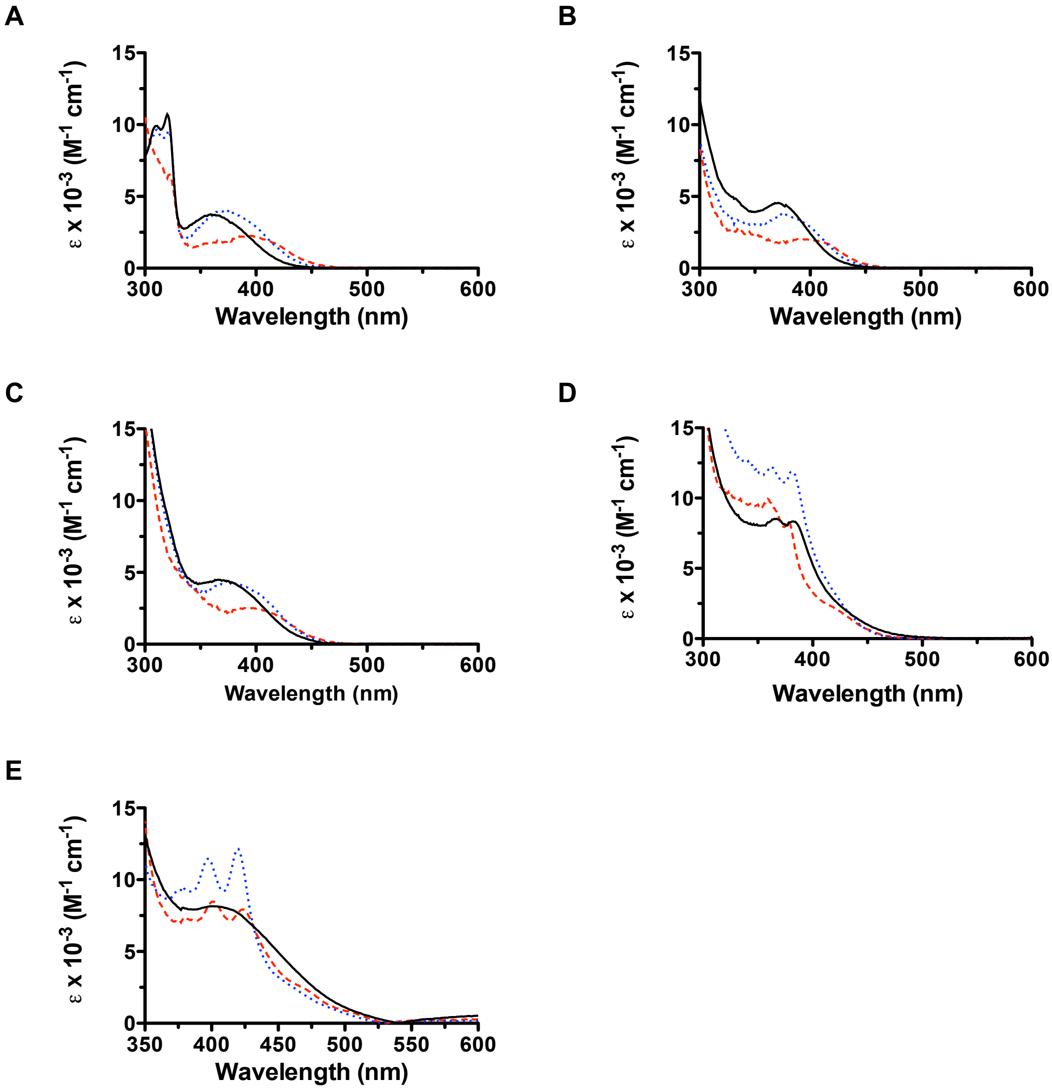

Figure S15. UV/Vis absorption changes of the MLCT band of complexes **1** (A), **2** (B), **3** (C), **4** (D), **5** (E) when prepared in different solvents: black (–): Tris-HCl buffer (5 mM, 50 mM NaCl, pH 7), blue (– –): CH_3CN, red (--): $CHCl_3$.

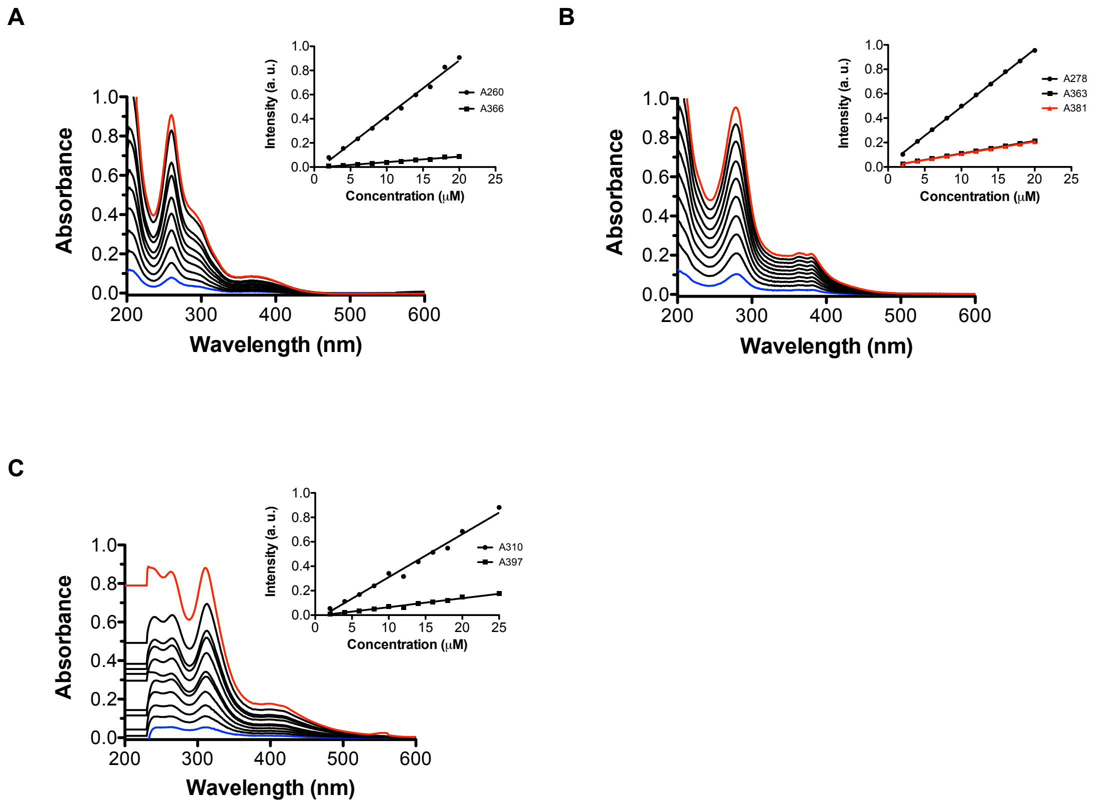

Figure S16. Absorption spectra of **3** (A), **4** (B), **5** (C) with increasing concentration from 2 (–) to 20 (–) μM in Tris-HCl buffer solution (5 mM, 50 mM NaCl, pH 7.0). Inset shows the absorption intensity at different absorption maxima against the complex concentration.

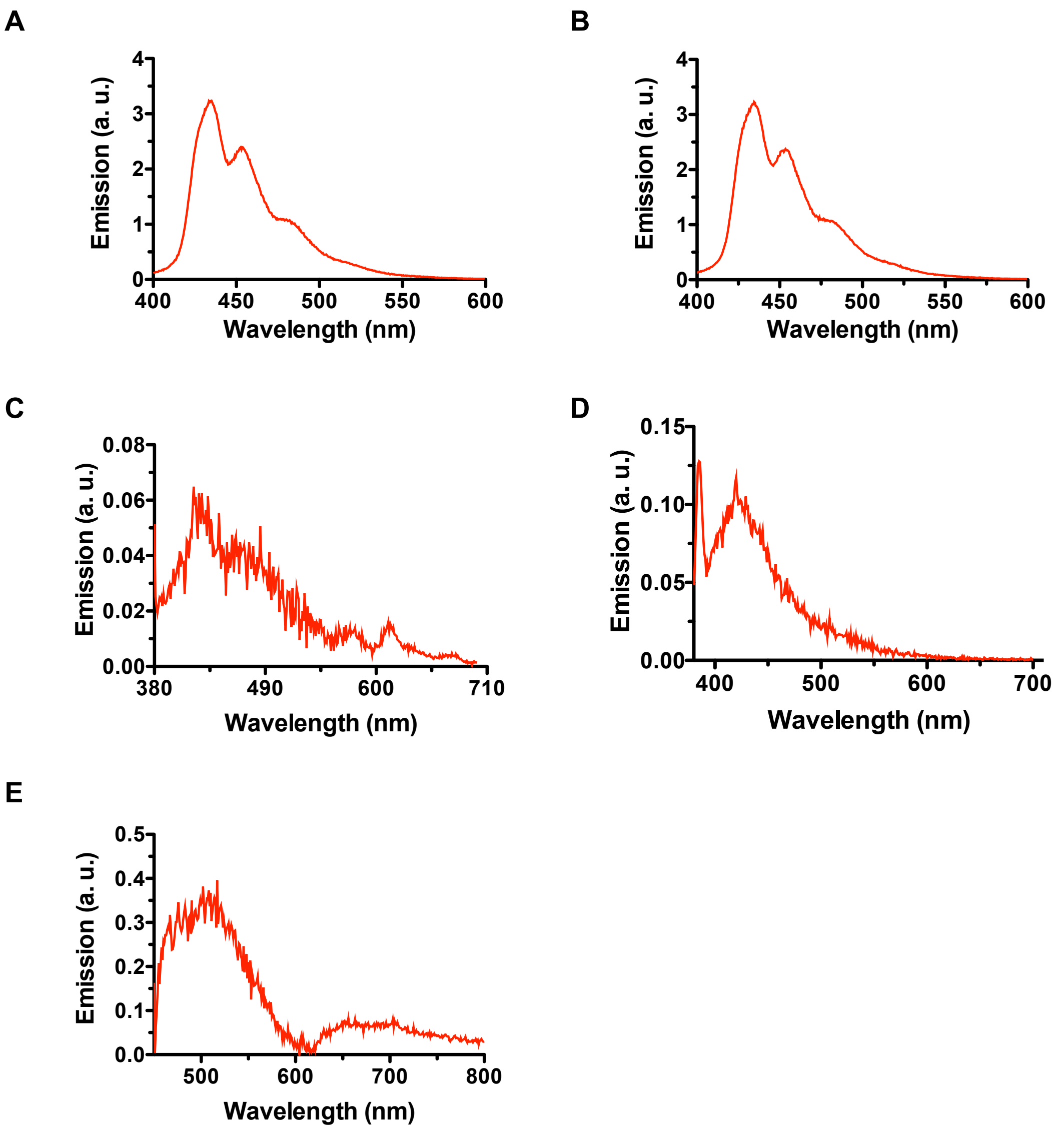

Figure S17. Emission spectra of complexes **1** (A), **2** (B) **3** (C), **4** (D), **5** (E) at 10 μM and 298 K in Tris-HCl buffer (5 mM, 50 mM NaCl, pH 7). λ_{ex} = 320, 330, 370, 367 and 430 nm for **1**–**5**, respectively. Note: 1% DMSO was used to ensure completely dissolution of complex **5**.

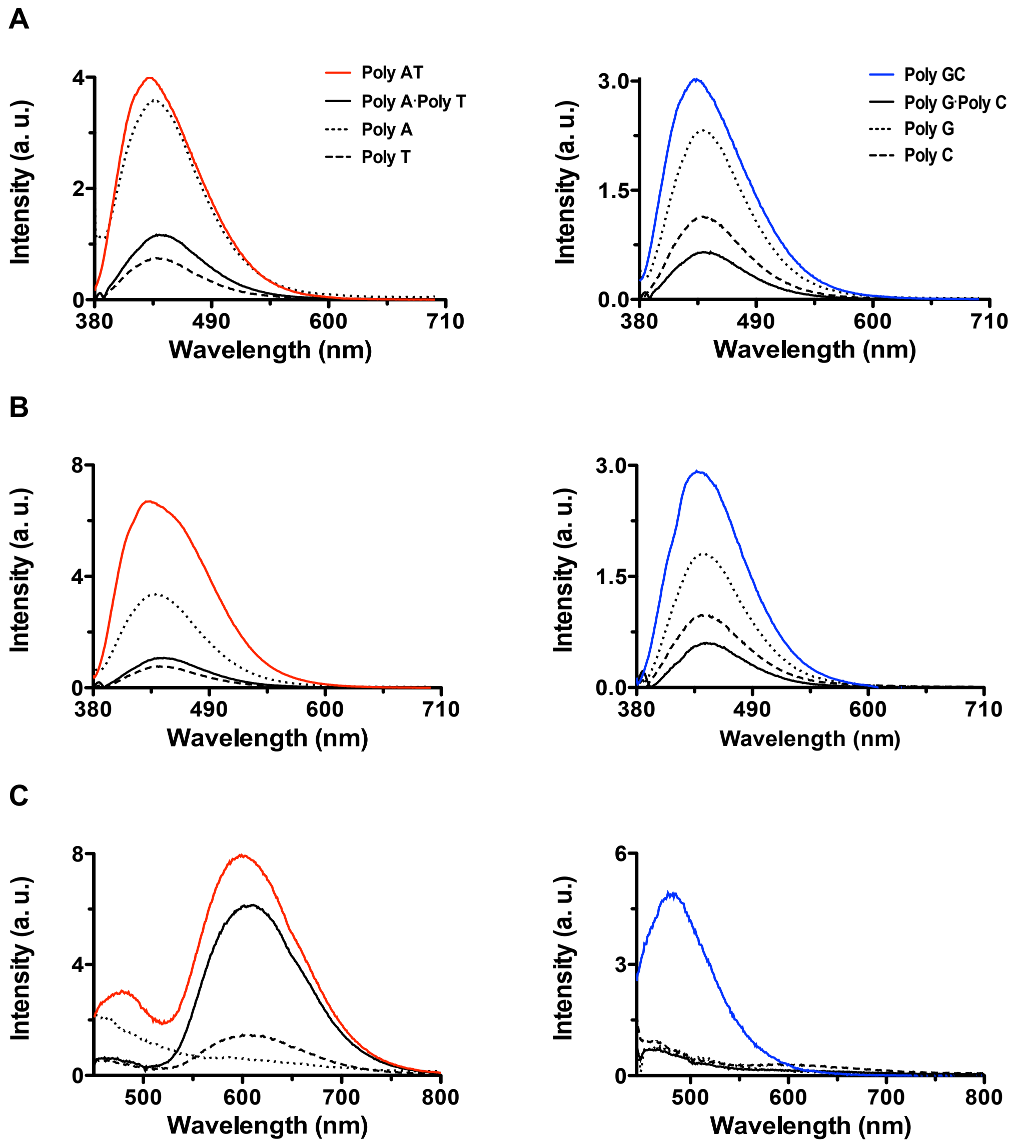

Figure S18. Emission spectra of complexes **3** (A), **4** (B), and **5** (C) at 10 μM in the presence of different types of DNAs (50 μM). The corresponding buffer used for each spectrum is specified in Table S2. λ_{ex} = 370, 367 and 430 nm for **3**–**5**, respectively.

Table S3. Luminescence area and λ_{max} of **3**.

Condition[a]	λ_{max} (nm)	Area	Ratio to buffer[b]
Buffer	420	5.8	1
CT DNA	456	10.25	1.8
poly GC	434	281.5	48.4
poly AT	438	361.8	62.2
poly A	437	345.2	59.3
poly T	438	66.1	11.4
poly G	440	208.4	35.8
poly C	440	98.6	16.9
poly A poly T	441	103.9	17.9
poly G poly C	447	55.8	9.6
inter G-quad	434	143.5	24.7
GC M	436	270.4	46.5
GC MM	435	265.5	45.7
AA M	438	290.7	50.0
AA MM	438	366.6	63.0

[a] For full experimental details see the Experimental Section and Table S2. [b] The luminescence area with the biomolecule compared to buffer alone.

Table S4. Luminescence area and λ_{max} of **4**.

Condition[a]	λ_{max} (nm)	Area	Ratio to buffer[b]
Buffer	458	9.3	1
CT DNA	420	8.31	0.90
poly GC	434	261.2	28.2
poly AT	431	684.2	73.9
poly A	440	318.1	34.4
poly T	441	65.9	7.1
poly G	443	156.2	16.9
poly C	441	86.7	9.4
poly A poly T	447	94.9	10.2
poly G poly C	446	50.5	5.5
inter G-quad	438	157.5	17.0
GC M	438	220.1	23.8
GC MM	438	216.1	23.3
AA M	438	179.8	19.4
AA MM	438	357.8	38.6

[a] For full experimental details see the Experimental Section and Table S2. [b] The luminescence area with the biomolecule compared to buffer alone.

Table S5. Luminescence area and λ_{max} of **5** with λ_{ex}= 430 nm.

Condition[a]	λ_{max} (nm)	Area	Ratio to buffer[b]
Buffer	445	34.6	1
CT DNA	445	76.5	2.2
poly GC	479	411.5	11.9
poly AT	599	1167	33.7
poly A	445	248.6	7.2
poly T	607	214.5	6.2
poly G	469	74.7	2.1
poly C	445	106.7	3.1
poly A poly T	611	773.5	22.4
poly G poly C	445	69.5	2.0
inter G-quad	445	273.8	7.9
GC M	445	211.2	6.1
GC MM	478	188.3	5.4
AA M	480	286.3	8.3
AA MM	445	242.6	7.0

[a] For full experimental details see the Experimental Section and Table S2. [b] The luminescence area with the biomolecule compared to buffer alone.

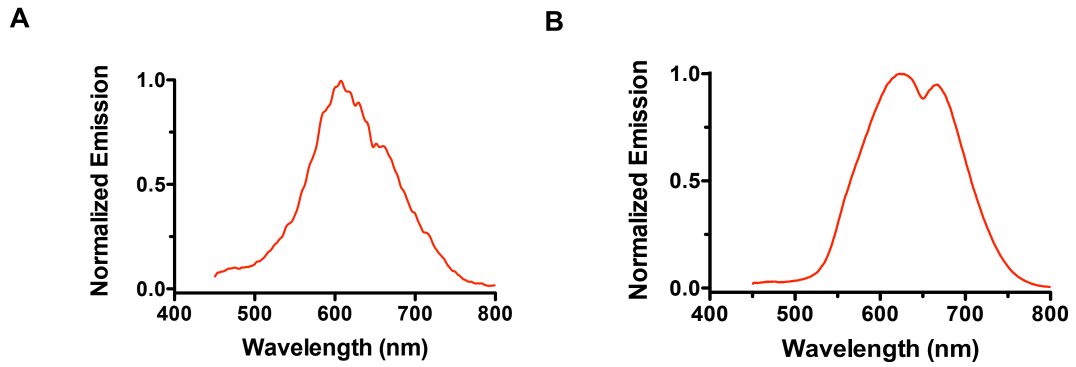

Figure S19. Emission spectra of (A) **5** (λ_{ex}= 430 nm, λ_{max}= 607 nm) and (B) dppn (λ_{ex}= 430 nm, λ_{max}= 623.5 and 667 nm) at 298 K in solid state.

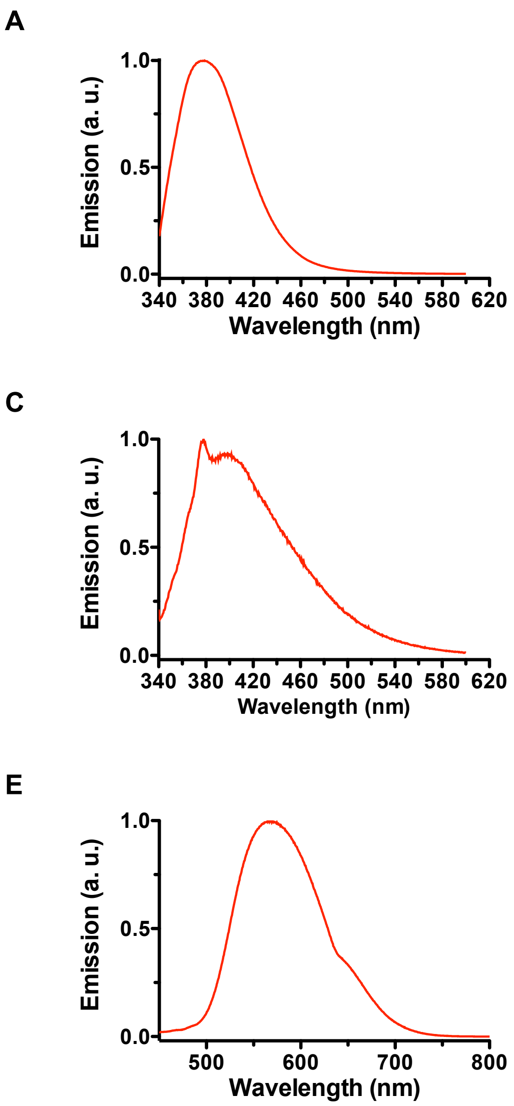

Figure S20. Emission (—) spectra of complexes **1** (A), **2** (B) **3** (C), **4** (D), **5** (E) at 10 μM and 298 K in ACN upon excitation at the low energy band. $\lambda_{em,max}$ = 378.5, 377, 418, 415 and 576 nm, respectively.

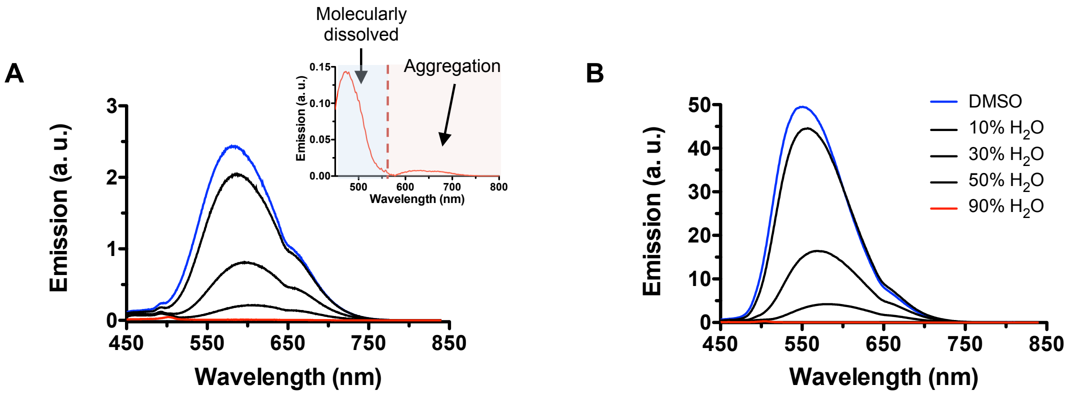

Figure S21. Emission spectra of complex **5** (A) and dppn (B) at 10 µM and 298 K in DMSO and different DMSO:H_2O mixtures upon excitation at λ_{ex} = 430 nm. The inset shows the final emission of **5** in the mixture DMSO:H_2O (10:90), similar to the emission of the complex in Tris-HCl buffer (Figure S11E). The concomitant addition of H_2O results in the quenching of the emission and a significant red-shift of the spectrum. At 90% of H_2O content, two emission peaks can be distinguished: one at 477 nm and another around 600 nm. This is consistent with the formation of H-bonding through the phenazine nitrogen atoms, which ultimately prevent the excimer formation in solution.

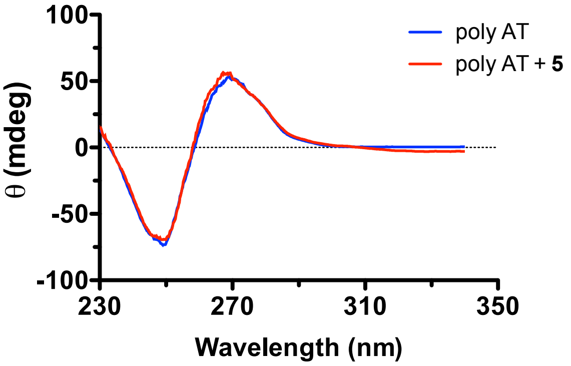

Figure S21. CD spectra of poly AT (50 µM) before and after 5 min incubation with **5** (10 µM). Note: Solution of **5** contains 0.33% DMSO.

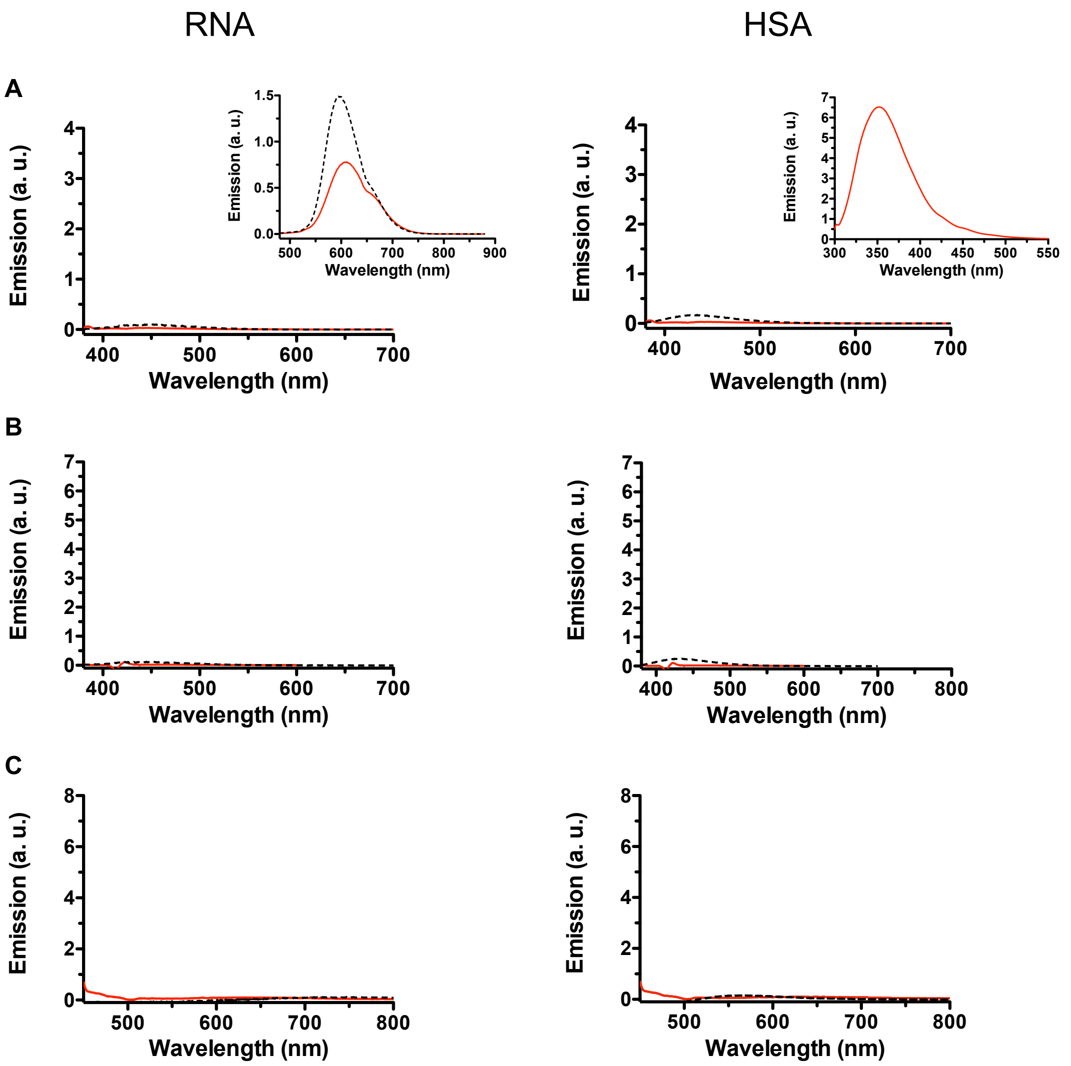

Figure S23. Emission spectra of **3** (A), **4** (B), **5** (C) in the absence (—) presence (- -) of 50 µM of RNA (left) or HSA (right) in Tris-HCl (5 mM, 50 mM NaCl, pH 7) buffer solution. Emission spectra were recorded using 10 µM concentration for **3**–**5**. Data was obtained with λ_{ex} = 370 nm, λ_{em} = 380–700 nm for **3**; λ_{ex} = 367 nm and λ_{em} = 377–700 for **4**; and λ_{ex} = 430 nm and λ_{em} = 445–800 nm for **5**. Note: 1% DMSO was used to ensure completely solubility of complex **5**. Insets show positive controls for RNA and HSA. For RNA, EtBr dissolved in Tris-HCl (5 mM, 50 mM NaCl, pH 7) buffer was used as control. The spectra of EtBr were collected in the absence (10 µM, - -) and presence of RNA (50 µM, —) using λ_{ex} = 450 nm, λ_{em} = 470–880 nm. The emission of the Trp residues in HSA was used as a positive control. Data was collected λ_{ex} = 295 nm; λ_{em} = 300-550 nm.

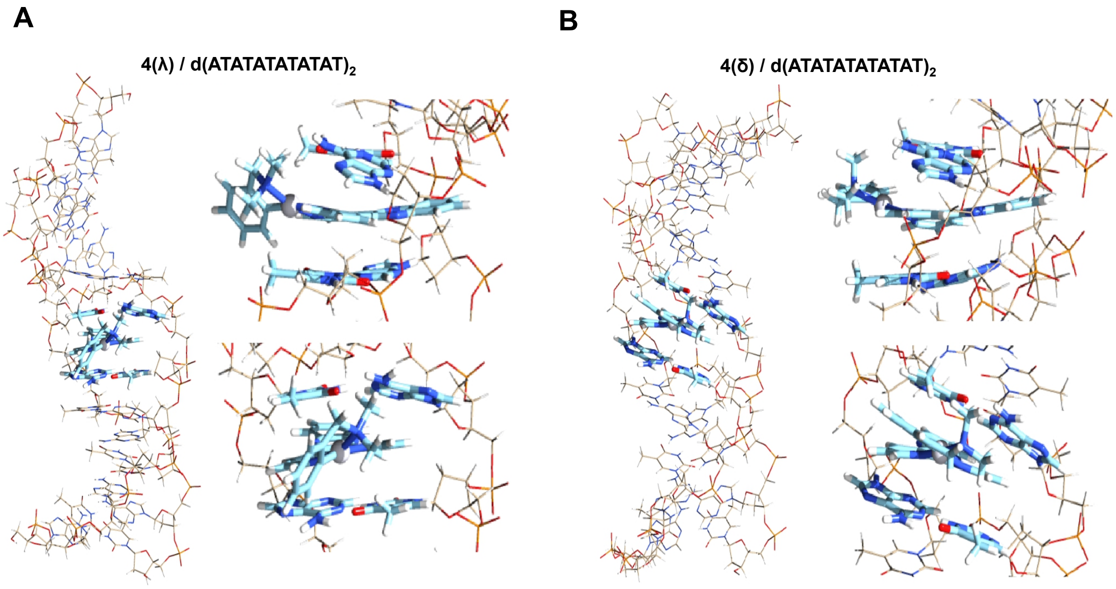

Figure S24. Front and side views of the optimized structure of the intercalation complexes between the two enantiomers of **4** with d(ATATATATATAT)$_2$, **λ** (A) and **δ** (B). High level and low level layers are shown as sticks and wires, respectively.

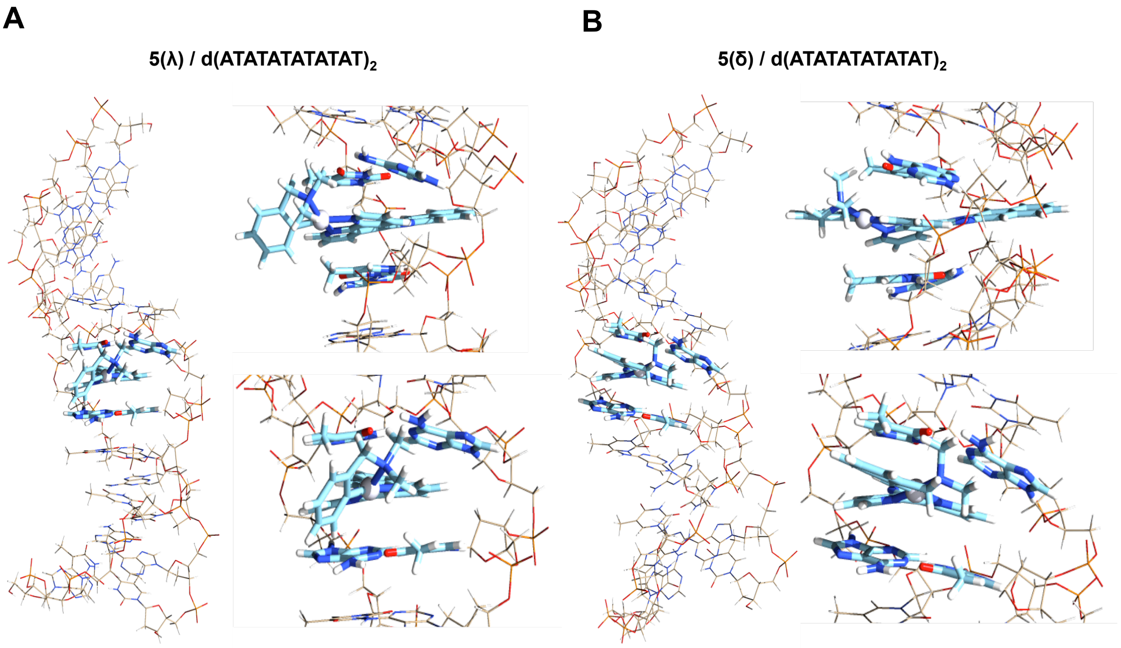

Figure S25. Front and side views of the optimized structure of the intercalation complexes between the two enantiomers of **5** with d(ATATATATATAT)$_2$, **λ** (A) and **δ** (B). High level and low level layers are shown as sticks and wires, respectively.

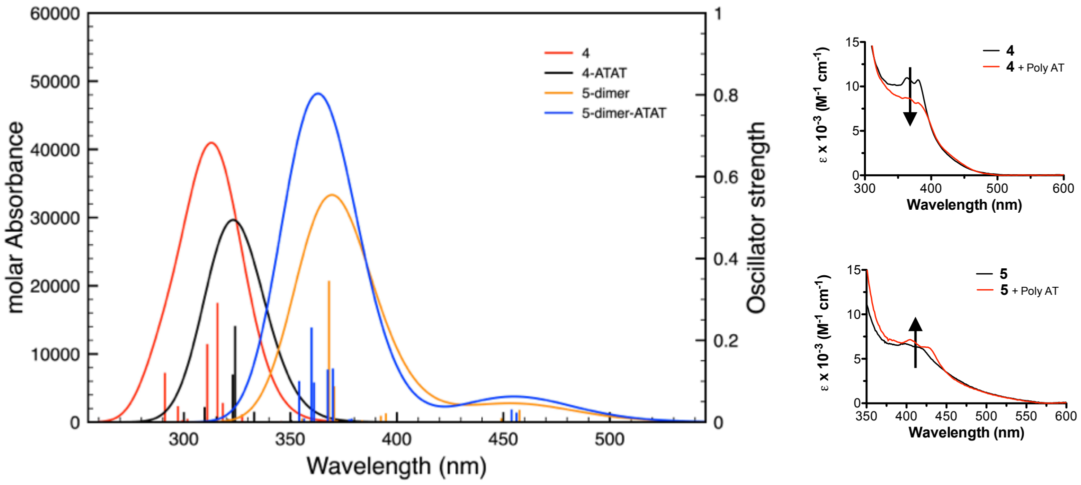

Figure S26. Absorption spectra calculated by TD-DFT on **4** and on the dimer of **5**, of the higher layer of DNA-intercalated **4** (see Figure S24A) and of the groove-binding dimer of **5** (see Figure 3A-3B), obtained by QM/MM calculations. Experimental UV/Vis are shown in the right.

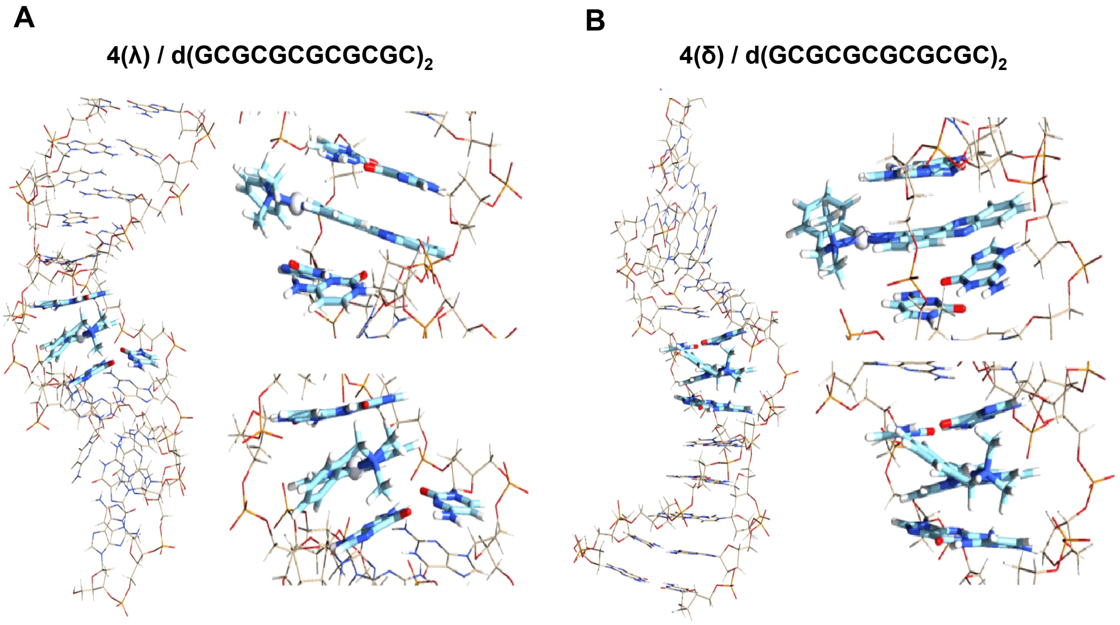

Figure S27. Front and side views of the optimized structure of the intercalation complexes between the two enantiomers of **4** with d(GCGCGCGCGCGC)$_2$, **λ** (A) and **δ** (B). High level and low level layers are shown as sticks and wires, respectively.

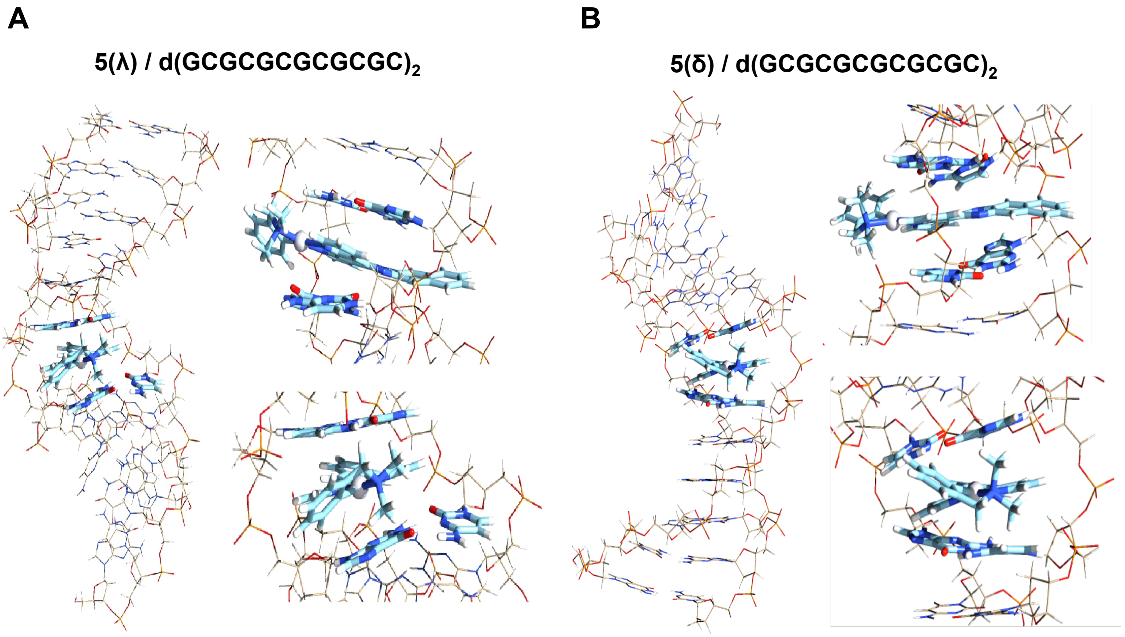

Figure S28. Front and side views of the optimized structure of the intercalation complexes between the two enantiomers of **5** with d(GCGCGCGCGCGC)$_2$, **λ** (A) and **δ** (B). High level and low level layers are shown as sticks and wires, respectively.

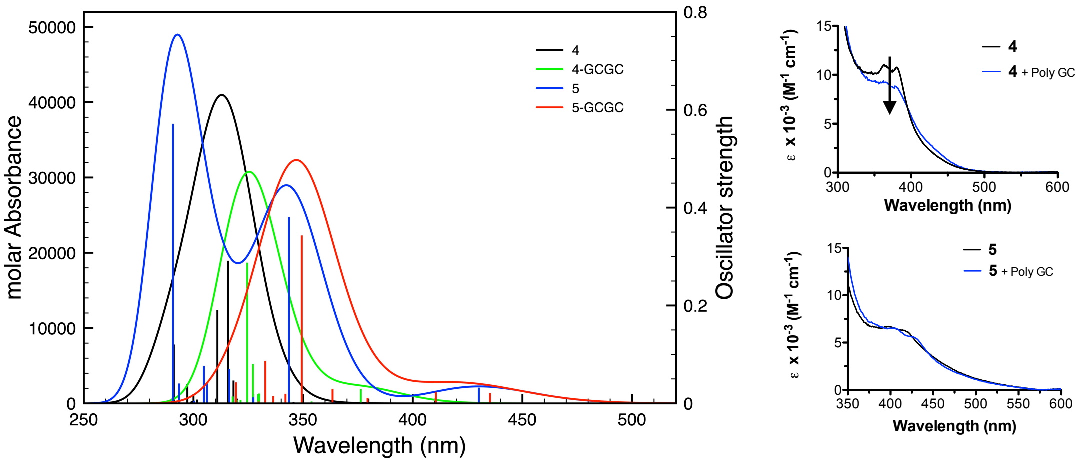

Figure S29. Absorption spectra calculated by TD-DFT on **4** and **5**, of the higher layer of DNA-intercalated **4** (see Figure S27A) and of DNA-intercalated **5** (see Figure S28A), obtained by QM/MM calculations. Experimental UV/Vis are shown in the right.

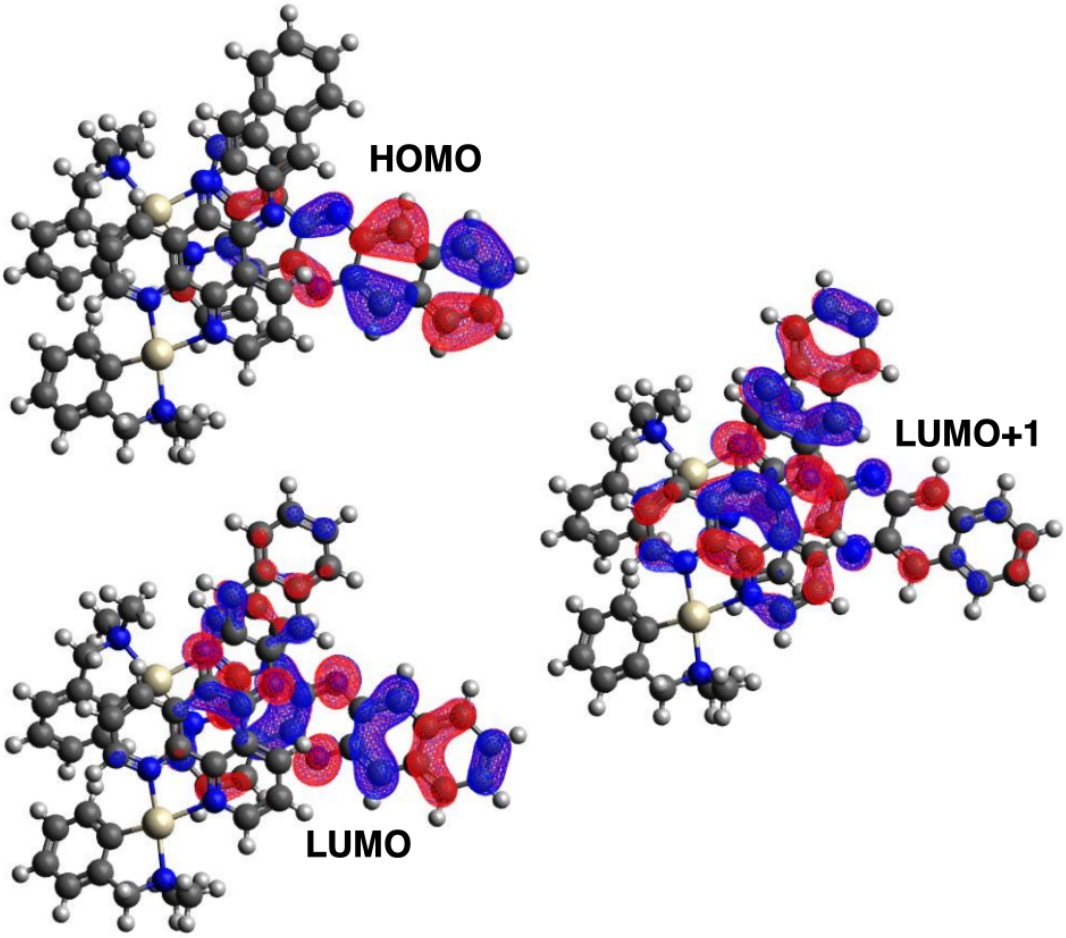

Figure S30. Molecular orbitals of the optimized structure for the dimer of **5** in the first excited state, involved in the lowest lying emission transition, which is a mixture of the HOMO → LUMO (87%) and HOMO → LUMO +1 (10%) transitions.

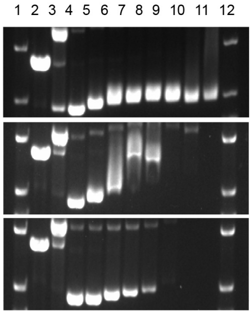

Figure S31. Agarose gels showing the dose response of **3** (upper), **4** (middle), **5** (lower) with 40 µg/mL pUC19 plasmid. Lane 1 and 12: DNA ladder; Lane 2: EcoRI; Lane 3: $Cu(OP)_2$; Lanes 4–11: 0, 7.8, 15.6, 31.3, 62.5, 125, 250, 500 µM of the corresponding metal complex. EcoRI and $Cu(OP)_2$ are used as controls for linear and relaxed circular DNA, respectively. EtBr was used to visualize the DNA. Note: the loss of EtBr signal for **4** and **5** was due to DNA precipitation at high complex concentrations

2. Crystal Data for Compound 4:

Compound code	Crystalization Solvents	Structure
(*N*,*N*-dimethylbenzylamine-κN, κC)(dipyrido[3,2-a:2',3'-c]phenazine)-platinum(II) nitrate hexane, dichloromethane	CH_2Cl_2/hexane	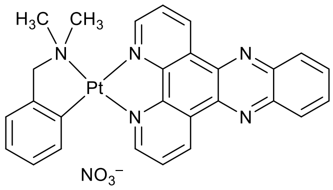

The single-crystal X-ray structure determination confirmed the anticipated molecular structures (Figure S23). Details of the structures solution and refinement are given in Table S6.

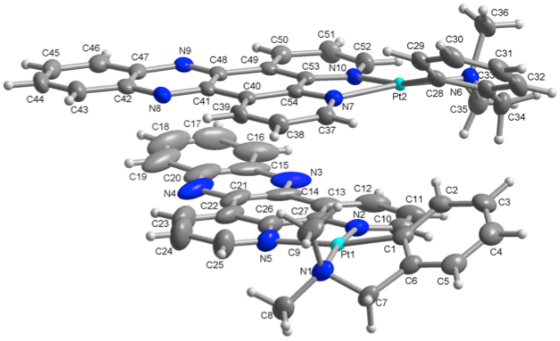

Figure S32. Fully atom labeled molecular structures of the two symmetry-independent cations of **4** (50% thermal ellipsoids). The nitrate anions are not shown.

Two symmetry-independent molecules of **4** or, more correctly, two identical chemical formula units were found here in the structural asymmetric unit[1] to give a Z' = 2 structure. Z' is defined as the number of formula units in the unit cell (here 4) divided by the number of independent general positions (here 2).[2] Different possibilities can give such Z' > 1 structures:[3] A structure which got stuck on its formation to a more stable form,[2] that is, a metastable crystal form[1,4] or strong and special supramolecular (e.g. hydrogen bonding, π-stacking) interactions between the two (or more) symmetry-independent units.[5] A high Z' is also obtained when the molecule has different conformations of very similar energy, with these conformations co-existing in the crystal.[6]

Here we ascribe the presence of two symmetry-independent molecules to π-stacking interactions between the two units (Figure S32, Table S7). Besides the cation-anion Coulomb interaction, the packing in the structure of **4** is organized by intermolecular π–π interactions[7] and less by C-H···π interactions[8] (Table S6, Figure S33). The π-stacking in **4** takes place between the electron-poor pyrazine and C_6-aromatic planes and also between the five-membered-platinum-chelate planes and C_6-aromatic planes (Figure S24). *Masui* had suggested an active electron delocalization within a metal-*N*-heterocyclic chelate ring in such a way that it could exhibit some degree of "metalloaromaticity".[9] Almost all ring systems of the dipyrido[3,2-a:2',3'-c]phenazine ligand are involved in significant π-stacking interactions (Table S7).

There are certainly additional weak C-H···O interactions which are evident to the found and refined nitrate anion. Since the second nitrate anion could not be fully located and refined, we refrained from further discussing these C-H···O interactions.

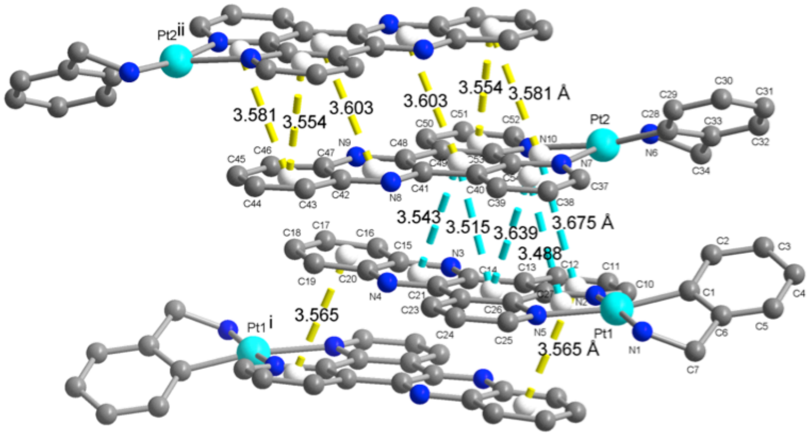

Figure S33. Section of the cation packing diagram of **4** showing significant π-stacking interactions (labelled with their centroid-centroid distances). See Table S7 for further details. π-Stacking between the two symmetry-independent molecules is depicted in cyan, otherwise in yellow. Hydrogen atoms and methyl groups are not shown for clarity. Symmetry transformations i = -X, 1-Y, 1-Z, ii = -X, 2-Y, 1-Z.

A top view of the two symmetry-independent cations of **4** seems to suggest the existence of a C_2-axis. This *pseudo*-C_2 axis is, however, not present because the five-membered platinum-*N*,*N*-dimethylbenzylamine chelate ring assume a chiral λ conformation at Pt1 and the enantiomeric δ conformation at Pt2 (Figure S32). Often these λ and δ enantiomeric conformations of a five-membered chelate ring have a low energy barrier for interconversion through a planar transition state and are only observed in the solid state. Here however, these λ and δ forms could also be present in solution as the steric repulsion between the β-C-H atoms on the *cis*-positioned aryl rings (Figure S34). Such a C-H repulsion to prevent racemization is similar as in the C_2-symmetric chiral 1,1'-binaphthyl compounds BINOL and BINAP.
At first sight one may also invoke the formation of λ and δ forms of the two symmetry-independent molecules as a cause of the Z' = 2 structure. However, in the centrosymmetric space group P-1, the mirror image λ and δ configurations would have been generated anyway by symmetry.

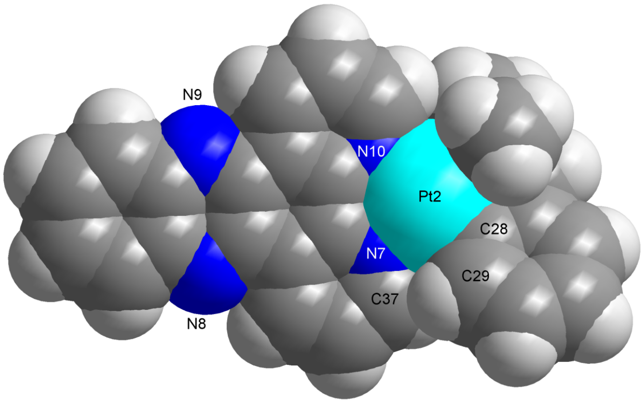

Figure S34. Top view in space-filling mode of one of the symmetry-independent cations to show the barrier for λ and δ interconversion due to steric repulsion between the β-C-H atoms C29-H and C37-H.

Noteworthy, the coordination sphere around the d^8 Pt(II) atom is not fully planar but the dihedral angle C-Pt-N(benzylamine) to C-Pt-C(phenazine) is 12.5(1)° at Pt1 and 13.4(1)° at Pt2. This deviation from planarity can also be traced to the steric repulsion between the β-C-H atoms on the benzyl and phenazine ring.
A four-coordinated nonplanar complex can also lead to metal-centered chirality. It occurs in non-planar systems with at least one asymmetric chelate ring A^B rendering a complex $M(A^B)_2$ or M(A^A)(A^B) chiral with C_2 symmetry for the $M(A^B)_2$ complex. The metal-centered configuration can be described using the Δ/Λ-nomenclature originally introduced for tris-chelate complexes.[10] The chelate ring is interpreted as a segment of a helix or screw along the (pseudo-)C_2 rotation axis (Scheme S1).[11] In Figure S32 the Pt1 atom has a Δ-configuration, the Pt2 atom the Λ-configuration. Hence in **4** each Pt molecule has a Δ-λ- or Λ-δ-configuration. Thus, the crystal presents a racemic mixture in agreement with the centrosymmetry of the P-1 space group.

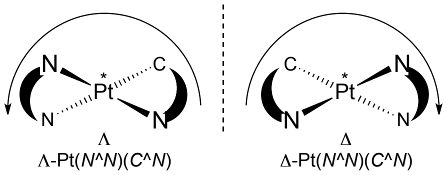

Scheme S1. Enantiomeric metal-centered absolute configuration of a pseudo-tetraedral, non-planar bischelate complex viewed down the "propeller blade" axis (perpendicular to the paper): Λ left-handed helicity, Δ right-handed helicity of the "propeller blades".[12]

Table S6. Crystal data and refinement parameters of **4**.

Crystal data

$2(C_{29}H_{22}N_3Pt)\cdot NO_3$ $(NO_3\cdot hexane\cdot 1.5CH_2Cl_2)^a$	$Z = 2$
$M_r = 1285.18$	$F(000) = 1250$
Triclinic, $P\bar{1}$	$D_x = 1.449$ Mg m^{-3}
$a = 13.6491$ (6) Å	Mo $K\alpha$ radiation, $\lambda = 0.71073$ Å
$b = 13.7262$ (7) Å	Cell parameters from 9901 reflections

c = 16.8800 (8) Å	θ = 2.6–30.5°
α = 86.245 (2)°	μ = 4.79 mm^{-1}
β = 69.010 (2)°	T = 173 K
γ = 88.025 (2)°	Prism, yellow
V = 2946.1 (2) $Å^3$	0.12 × 0.08 × 0.06 mm

Data Collection

Bruker D8 QUEST CCD diffractometer	10455 reflections with $I > 2\sigma(I)$
Radiation source: fine-focus sealed tube	R_{int} = 0.040
ω and ϕ scans	θ_{max} = 25.9°, θ_{min} = 2.0°
Absorption correction: multi-scan (*SADABS*; Sheldrick, 1996)	h = -16→16
T_{min} = 0.559, T_{max} = 0.746	k = -16→16
149360 measured reflections	l = -20→20
11401 independent reflections	

Refinement

Refinement on F^2	0 restraints
Least-squares matrix: full	Hydrogen site location: inferred from neighbouring sites
$R[F^2 > 2\sigma(F^2)]$ = 0.0214<br>$R((F^2$, all data) = 0.252	H-atom parameters constrained
$wR[F^2 > 2\sigma(F^2)]$ = 0.0487<br>$wR(F^2$, all data) = 0.0498	$w = 1/[\sigma^2(F_o^2) + (0.0183P)^2 + 7.1757P]$<br>where $P = (F_o^2 + 2F_c^2)/3$
S = 1.05	$(\Delta/\sigma)_{max}$ = 0.002
11396 reflections	$\Delta\rangle_{max}$ = 1.30 e $Å^{-3}$
635 parameters	$\Delta\rangle_{min}$ = -0.69 e $Å^{-3}$

[a]One disordered nitrate anion, the disordered hexane and 1.5 disordered CH_2Cl_2 molecules could be localized but not satisfactorily refined. Hence their electron count was treated with the SQUEEZE option in Platon.[13]

Table S7. Packing Analysis for **4** for possible π-π interactions.[a]

==

Analysis of Short Ring-Interactions with Cg-Cg Distances < 6.0 Angstrom and Beta < 60.0Deg.

==

- Cg(I) = Plane number I (= ring number in () above)
- Alpha = Dihedral Angle between Planes I and J (Deg)
- Beta = Angle Cg(I)-->Cg(J) or Cg(I)-->Me vector and normal to plane I (Deg)
- Gamma = Angle Cg(I)-->Cg(J) vector and normal to plane J (Deg)
- Cg-Cg = Distance between ring Centroids (Ang.)
- CgI_Perp = Perpendicular distance of Cg(I) on ring J (Ang.)
- CgJ_Perp = Perpendicular distance of Cg(J) on ring I (Ang.)
- Slippage = Distance between Cg(I) and Perpendicular Projection of Cg(J) on Ring I (Ang).
- P,Q,R,S = J-Plane Parameters for Carth. Coord. (Xo, Yo, Zo)

Cg(I) Res(I)	Cg(J) [ARU(J)]	Cg-Cg	Alpha	Beta	Gamma	CgI_Perp	CgJ_Perp
Cg(2) [1] ->	Cg(23) [1555.02]	3.4884(16)	4.09(13)	13.63	16.56	-3.3437(11)	3.3901(11)
Cg(3) [1] ->	Cg(8) [2566.01]	3.565(2)	5.52(18)	20.25	16.87	-3.4114(14)	-3.3444(18)
Cg(3) [1] ->	Cg(22) [1555.02]	3.6750(17)	7.84(14)	19.00	11.70	-3.5986(13)	3.4747(11)
Cg(4) [1] ->	Cg(27) [1555.02]	3.5426(18)	4.13(15)	19.87	22.86	-3.2643(14)	3.3317(11)
Cg(7) [1] ->	Cg(23) [1555.02]	3.6389(16)	4.37(13)	22.24	20.85	-3.4005(11)	3.3682(11)
Cg(7) [1] ->	Cg(27) [1555.02]	3.5150(17)	2.61(13)	18.65	16.69	-3.3670(12)	3.3304(11)
Cg(8) [1] ->	Cg(3) [2566.01]	3.565(2)	5.52(18)	16.87	20.25	-3.3443(18)	-3.4114(14)
Cg(22) [2] ->	Cg(3) [1555.01]	3.6750(17)	7.84(14)	11.70	19.00	3.4748(11)	-3.5986(14)
Cg(22) [2] ->	Cg(28) [2576.02]	3.5813(16)	3.59(13)	18.74	22.32	3.3131(11)	3.3916(12)
Cg(23) [2] ->	Cg(2) [1555.01]	3.4884(16)	4.09(13)	16.56	13.63	3.3901(11)	-3.3437(11)
Cg(23) [2] ->	Cg(7) [1555.01]	3.6388(16)	4.37(13)	20.85	22.24	3.3681(11)	-3.4004(11)
Cg(24) [2] ->	Cg(27) [2576.02]	3.6030(16)	1.28(13)	23.94	24.03	3.2907(11)	3.2931(11)
Cg(25) [2] ->	Cg(28) [2576.02]	3.5543(16)	2.79(13)	19.16	19.04	3.3597(11)	3.3573(12)
Cg(27) [2] ->	Cg(4) [1555.01]	3.5426(18)	4.13(15)	22.86	19.87	3.3318(11)	-3.2642(14)
Cg(27) [2] ->	Cg(7) [1555.01]	3.5150(17)	2.61(13)	16.69	18.65	3.3304(11)	-3.3670(12)
Cg(27) [2] ->	Cg(24) [2576.02]	3.6030(16)	1.28(13)	24.03	23.94	3.2931(11)	3.2907(11)
Cg(28) [2] ->	Cg(22) [2576.02]	3.5813(16)	3.59(13)	22.32	18.74	3.3916(12)	3.3131(10)
Cg(28) [2] ->	Cg(25) [2576.02]	3.5542(16)	2.79(13)	19.04	19.16	3.3573(12)	3.3597(11)

Min or Max 3.488

[2566] = -X,1-Y,1-Z
[2665] = 1-X,1-Y,-Z
[1555] = X,Y,Z
[1555] = X,Y,Z
[2576] = -X,2-Y,1-Z
[2575] = -X,2-Y,-Z

[a]The Table presents a selection of the Cg-Cg distances calculated by PLATON,[13] here chosen according to the criteria of centroid-centroid contacts (< 3.8 Å), near parallel ring planes (alpha < 10° to ~0° or even exactly 0° by symmetry), small slip angles (β, γ < 25°) (Scheme S1).
Cyan highlights significant interactions between the two symmetry-independent molecules of the asymmetric unit. Interactions highlighted in yellow are to symmetry-related neighboring molecules. The highlighted interactions are depicted in Figure S33.

The Cg(I) refer to the Ring Centre-of-Gravity numbers with atoms#
Cg(2) = Pt1-N2-N5-C26-C27
Cg(3) = N2-C10-C11-C12-C13-C27
Cg(4) = N3-N4-C14-C15-C20-C21
Cg(7) = C13-C14-C21-C22-C26-C27
Cg(8) =C15-C16-C17-C18-C19-C20
Cg(22) = Pt2-N7-N10-C53-C54
Cg(23) = N7-C37-C38-C39-C40-C54
Cg(24) = N8-N9-C41-C42-C47-C48
Cg(25) = N10-C49-C50-C51-C52-C53
Cg(27) = C40-C41-C48-C49-C53-C54
Cg(28) = C42-C43-C44-C45-C46-C47

===

Analysis of X-H...Cg(Pi-Ring) Interactions (H..Cg < 3.0 Ang. - Gamma < 30.0 Deg)

===

- Cg(J) = Center of gravity of ring J (Plane number above)
- H-Perp = Perpendicular distance of H to ring plane J
- Gamma = Angle between Cg-H vector and ring J normal
- X-H..Cg = X-H-Cg angle (degrees)
- X..Cg = Distance of X to Cg (Angstrom)
- X-H, Pi = Angle of the X-H bond with the Pi-plane (i.e.' Perpendicular = 90 degrees, Parallel = 0 degrees)

X--H(I) Res(I) Cg(J) [ARU(J)]	H..Cg	H-Perp	Gamma	X-H..Cg	X..Cg	X-H,Pi
C(7) -H(7B) [1] -> Cg(6) [2665.01]	2.47	2.47	4.11	162	3.427(3)	68
Min or Max	2.470	2.466	4.11	162.00	3.427	68.00

[2665] = 1-X,1-Y,-Z
Cg(6) = C1-C2-C3-C4-C5-C6

===

Significant π-stackings show rather short centroid-centroid contacts (< 3.8 Å), near parallel ring planes (alpha < 10° to ~0° or even exactly 0° by symmetry), small slip angles (β, γ < 25°) and vertical displacements (slippage < 1.5 Å), which translate into a sizable overlap of the aryl-plane areas (Scheme S2).[14] Significant intermolecular C-H···π contacts are less than 2.7 Å for the (C-)H···ring centroid distances with H-perp below 2.6-2.7 Å and C-H··Cg > 145°.[15]

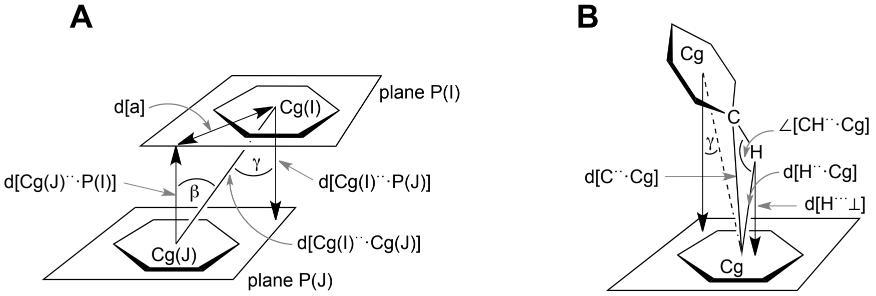

Scheme S2. Graphical presentation of the parameters used for the description of (A) π–π stacking and (B) CH-π interactions.

Table S8. Selected bond distances and angles (Å, °) for **4**.

Pt1—C1	1.995 (3)	Pt2—C28	1.998 (3)
Pt1—N2	2.024 (3)	Pt2—N7	2.022 (2)
Pt1—N1	2.085 (3)	Pt2—N6	2.086 (3)
Pt1—N5	2.147 (3)	Pt2—N10	2.150 (3)
C1—Pt1—N2	97.91 (12)	C28—Pt2—N7	97.48 (11)
C1—Pt1—N1	80.81 (12)	C28—Pt2—N6	80.86 (11)
N2—Pt1—N1	172.31 (10)	N7—Pt2—N6	173.18 (10)
C1—Pt1—N5	170.69 (11)	C28—Pt2—N10	168.94 (11)
N2—Pt1—N5	79.06 (11)	N7—Pt2—N10	79.40 (10)
N1—Pt1—N5	103.36 (11)	N6—Pt2—N10	103.45 (10)

3. Crystal Data for Compound 2

Compound code	Crystalization Solvents	Structure
(*N*,*N*-dimethylbenzylamine-κN, κC)(1,10 phenanthroline)-platinum(II) nitrate trihydrate	D_2O	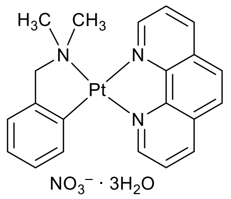

The single-crystal X-ray structure determination confirmed the anticipated molecular structures (Figure S35). Details of the structures solution and refinement are given in Table S9. The (*N*,*N*-dimethylbenzylamine-κN, κC)(1,10-phenanthroline)platinum(II) cation and nitrate anion crystallize with three water molecules per formula unit. The nitrate anion is part of the hydrogen-bonding interactions of the water molecules of crystallization (cf. Figure S35 and Figure S36A).

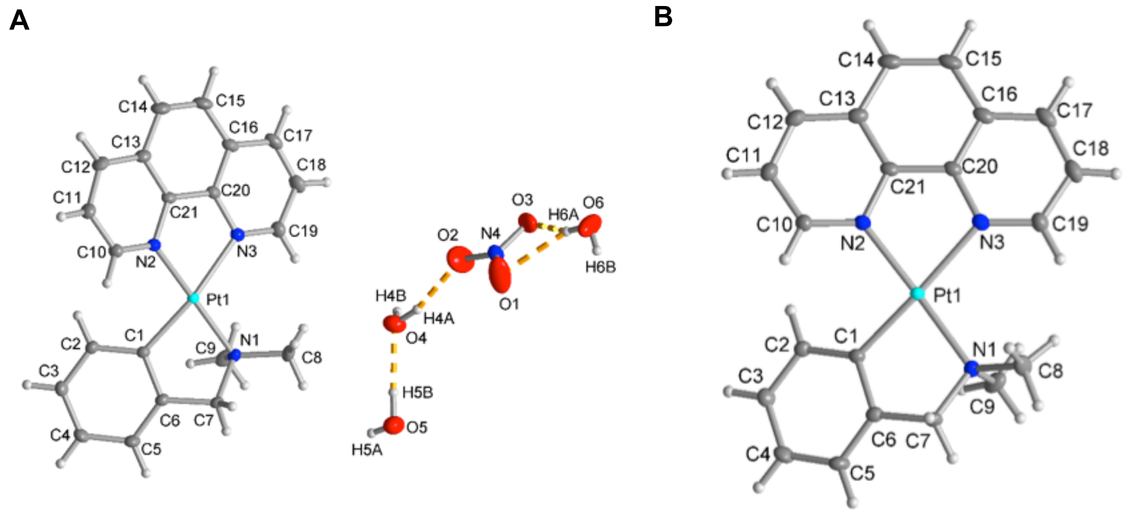

Figure S35. Molecular structure of (A) the asymmetric unit and (B) the cation only of **2** (50% thermal ellipsoids). In (A) the hydrogen bonding scheme is indicated as orange dashed lines. In (B) the nitrate anion and water molecules or crystallization are not shown. For bond distances and angles see Table S11. For details of hydrogen bonding interactions see Table S12.

The hydrophobic/non-polar cation and the hydrophilic/polar nitrate anion with the crystal water molecules are separately organized in strands along the *c* direction (Figure S36).

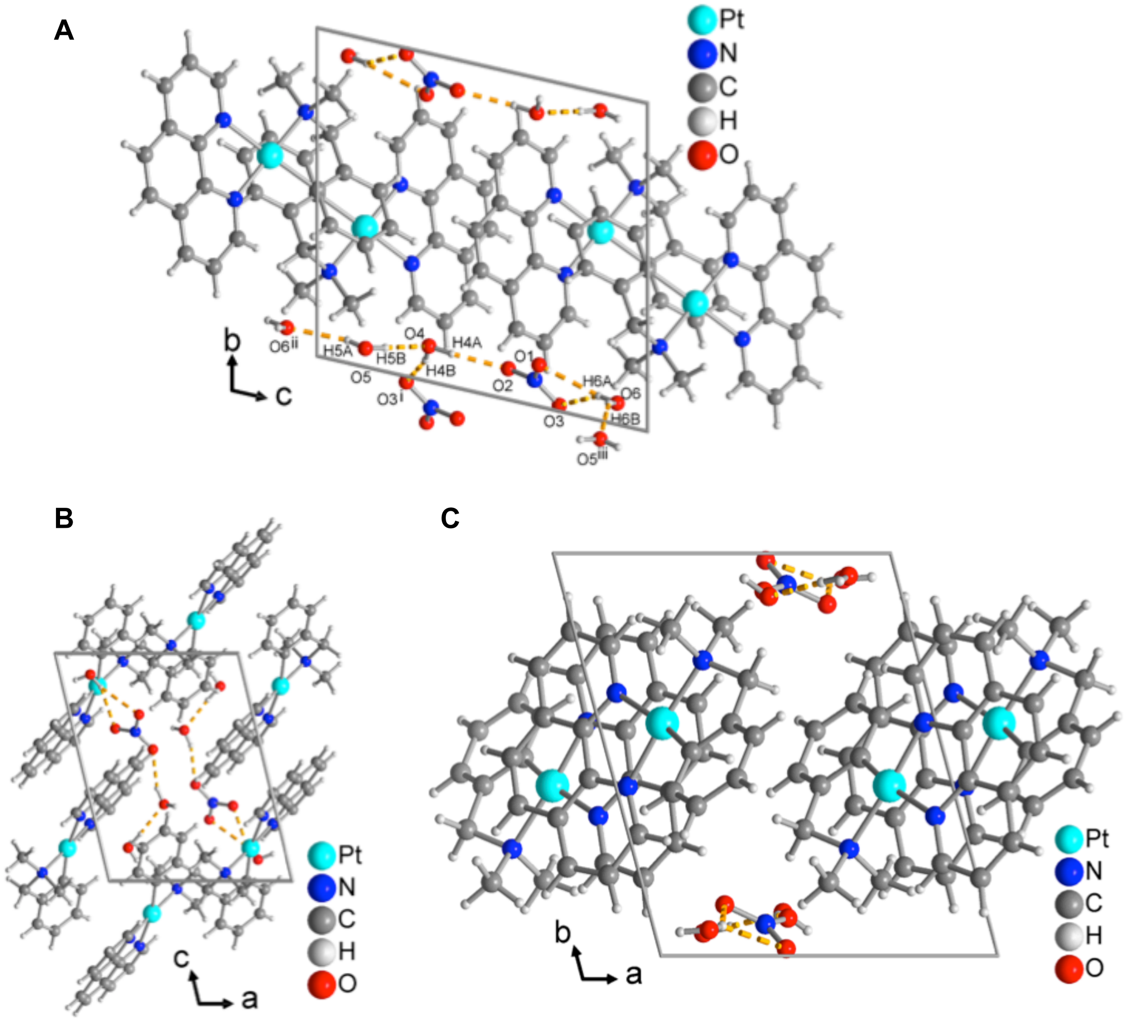

Figure S36. Sections of the packing diagrams along *a*, *b* and *c* in the structure of **2** to show the separation of non-polar and polar building blocks. The packing diagram in (A) depicts also the full O-H...O hydrogen bonding scheme. For details of hydrogen bonding interactions see Table S12.

As discussed for complex **4**, compound **2** crystallizes in the P-1 space group and the packing in the structure is organized by intermolecular π–π interactions (Figure S37, Table S10) and less by C-H···π interactions (Table S12). The main difference is that there is only one symmetry-independent molecule in **2**, *i.e.* the λ enantiomer.

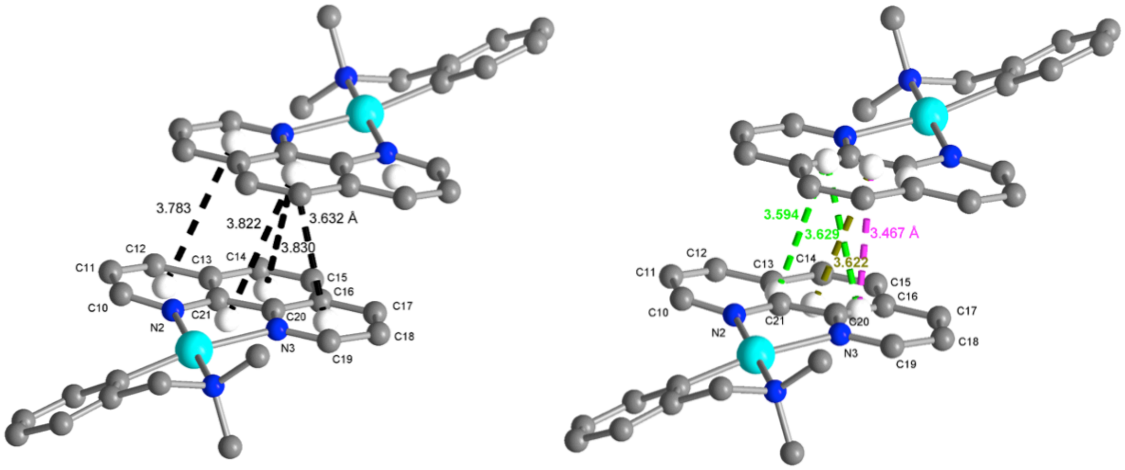

Figure S37. Section of the cation packing diagram of **2** showing selected significant π-stacking interactions (labelled with their centroid-centroid distances). See Table S10 for further details. Centroid-centroid contacts between the six-membered rings are depicted in black, between 10-membered rings in green, between the full 14-membered phenanthroline rings in dark-yellow and between a 10-to-14 membered ring in pink (cf. color code in Table S10). Additinal π-contacts between 6-to-10 and 6-to-14 membered rings are not shown but listed in Table S10. Hydrogen atoms and methyl groups are not shown for clarity.

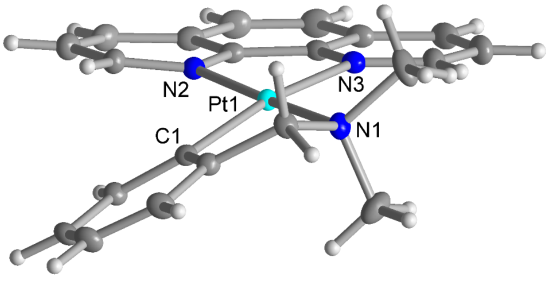

Figure S38. Front view of the cation of **2**.

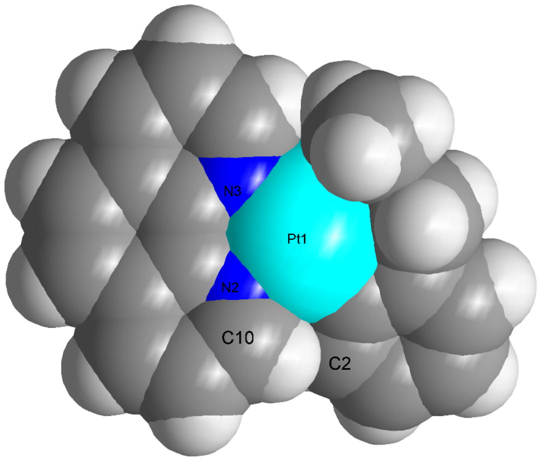

Figure S39. Top view in space-filling mode of the cation to show the barrier for λ and δ interconversion due to steric repulsion between the β-C-H atoms C2-H and C10-H.

Table S9. Crystal data and refinement parameters of **2**.

Crystal data

$C_{21}H_{20}N_3Pt{\cdot}NO_3{\cdot}3(H_2O)$	$Z = 2$
$M_r = 625.55$	$F(000) = 612$
Triclinic, $P\bar{1}$	$D_x = 1.904$ Mg m^{-3}
$a = 9.0700$ (5) Å	Mo $K\alpha$ radiation, $\lambda = 0.71073$ Å
$b = 11.1917$ (7) Å	Cell parameters from 9679 reflections
$c = 11.4941$ (7) Å	$\theta = 2.4$–$30.6°$
$\alpha = 99.945$ (2)°	$\mu = 6.48$ mm^{-1}
$\beta = 100.661$ (2)°	$T = 100$ K
$\gamma = 102.604$ (2)°	Block, yellow
$V = 1091.01$ (11) Å^3	0.13 × 0.06 × 0.05 mm

Data collection

Bruker D8 QUEST CCD diffractometer	6439 reflections with $I > 2\sigma(I)$
Radiation source: fine-focus sealed tube	$R_{int} = 0.027$
ω and ϕ scans	$\theta_{max} = 30.6°$, $\theta_{min} = 1.9°$
Absorption correction: multi-scan (*SADABS*; Sheldrick, 1996)	$h = -12 \rightarrow 12$
$T_{min} = 0.538$, $T_{max} = 0.746$	$k = -16 \rightarrow 16$
116751 measured reflections	$l = -16 \rightarrow 16$
6683 independent reflections	

Refinement

Refinement on F^2	0 restraints
Least-squares matrix: full	Hydrogen site location: mixed
$R[F^2 > 2\sigma(F^2)] = 0.0150$<br>$R(F^2)$ (all data) = 0.0163	H atoms treated by a mixture of independent and constrained refinement
$wR[F^2 > 2\sigma(F^2)] = 0.0358$<br>$wR(F^2)$ (all data) = 0.0364	$w = 1/[\sigma^2(F_o^2) + (0.0164P)^2 + 1.4328P]$<br>where $P = (F_o^2 + 2F_c^2)/3$
$S = 1.091$	$(\Delta/\sigma)_{max} = 0.002$
6683 reflections	$\Delta\rangle_{max} = 2.22$ e Å^{-3}
309 parameters	$\Delta\rangle_{min} = -0.90$ e Å^{-3}

Table S10. Packing Analysis for **2** for possible – and significant – π–π and C-H-π interactions.[a]

===

Analysis of Short Ring-Interactions with Cg-Cg Distances < 6.0 Angstrom and Beta < 60.0Deg.

===

- Cg(I) = Plane number I (= ring number in () above)
- Alpha = Dihedral Angle between Planes I and J (Deg)
- Beta = Angle Cg(I)-->Cg(J) or Cg(I)-->Me vector and normal to plane I (Deg)
- Gamma = Angle Cg(I)-->Cg(J) vector and normal to plane J (Deg)
- Cg-Cg = Distance between ring Centroids (Ang.)
- CgI_Perp = Perpendicular distance of Cg(I) on ring J (Ang.)
- CgJ_Perp = Perpendicular distance of Cg(J) on ring I (Ang.)
- Slippage = Distance between Cg(I) and Perpendicular Projection of Cg(J) on Ring I (Ang).
- P,Q,R,S = J-Plane Parameters for Carth. Coord. (Xo, Yo, Zo)

Cg(I) Res(I) Cg(J) [ARU(J)]	Cg-Cg	slippage	Alpha	Beta	Gamma	CgI_Perp	CgJ_Perp
Cg(2) [1] -> Cg(6) [2766.01]	3.8223(11)	1.736	5.32(8)	27.0	22.4	-3.5347(7)	-3.4055(8)
Cg(3) [1] -> Cg(4) [2766.01]	3.7831(11)	1.451	2.99(10)	22.5	24.3	-3.4483(8)	-3.4939(9)
Cg(4) [1] -> Cg(3) [2766.01]	3.7831(11)	1.556	2.99(10)	24.3	22.5	-3.4940(9)	-3.4482(8)
Cg(4) [1] -> Cg(6) [2766.01]	3.6318(12)	1.091	0.82(9)	17.5	16.8	-3.4771(9)	-3.4640(8)
Cg(4) [1] -> Cg(7) [2766.01]	3.5024(10)	0.599	0.95(8)	9.9	10.4	-3.4454(9)	-3.4507(6)
Cg(4) [1] -> Cg(9) [2766.01]	3.7190(10)	1.390	0.27(8)	21.9	22.1	-3.4457(9)	-3.4494(5)
Cg(6) [1] -> Cg(2) [2766.01]	3.8225(11)	1.455	5.32(8)	22.4	27.0	-3.4056(8)	-3.5347(7)
Cg(6) [1] -> Cg(4) [2766.01]	3.6319(12)	1.049	0.82(9)	16.8	17.5	-3.4640(8)	-3.4771(9)
Cg(6) [1] -> Cg(6) [2766.01]	3.8306(11)	1.607	0.00(9)	24.8	24.8	-3.4774(8)	-3.4773(8)
Cg(6) [1] -> Cg(8) [2766.01]	3.5334(10)	0.623	0.38(8)	10.2	10.4	-3.4748(8)	-3.4780(6)
Cg(6) [1] -> Cg(9) [2766.01]	3.6195(10)	1.075	1.08(7)	17.3	16.6	-3.4692(8)	-3.4561(5)
Cg(7) [1] -> Cg(4) [2766.01]	3.5023(10)	0.629	0.95(8)	10.4	9.9	-3.4507(6)	-3.4454(9)
Cg(7) [1] -> Cg(8) [2766.01]	3.5939(9)	0.920	1.34(6)	14.8	16.2	-3.4520(6)	-3.4741(6)
Cg(8) [1] -> Cg(6) [2766.01]	3.5333(10)	0.641	0.38(8)	10.4	10.2	-3.4780(6)	-3.4747(8)
Cg(8) [1] -> Cg(7) [2766.01]	3.5938(9)	1.000	1.34(6)	16.2	14.8	-3.4740(6)	-3.4520(6)
Cg(8) [1] -> Cg(8) [2766.01]	3.6294(9)	1.051	0.03(6)	16.8	16.8	-3.4740(6)	-3.4740(6)
Cg(8) [1] -> Cg(9) [2766.01]	3.4671(9)	0.252	0.73(5)	4.2	4.1	-3.4583(6)	-3.4579(5)
Cg(9) [1] -> Cg(4) [2766.01]	3.7190(10)	1.399	0.27(8)	22.1	21.9	-3.4494(5)	-3.4457(9)
Cg(9) [1] -> Cg(6) [2766.01]	3.6194(10)	1.032	1.08(7)	16.6	17.3	-3.4561(5)	-3.4692(8)
Cg(9) [1] -> Cg(8) [2766.01]	3.4671(9)	0.247	0.73(5)	4.1	4.2	-3.4579(5)	-3.4583(6)
Cg(9) [1] -> Cg(9) [2766.01]	3.6215(8)	1.083	0.03(4)	17.4	17.4	-3.4558(5)	-3.4558(5)
Min	3.467		0.0	4.1			

[2766] = 2-X,1-Y,1-Z

[2665] = 1-X,1-Y,-Z

[2765] = 2-X,1-Y,-Z
[2866] = 3-X,1-Y,1-Z

[a]The Table presents a selection of the Cg-Cg distances calculated by PLATON,[7] here chosen according to the criteria of centroid-centroid contacts (<3.8 Å), near parallel ring planes (alpha < 10° to ~0° or even exactly 0° by symmetry), small slip angles (β, γ < 25°) (Scheme S1).
Interactions highlighted in color are the unique interactions between the two symmetry-related neighboring molecules. Selected highlighted interactions are depicted in Figure S37.

The Cg(I) refer to the Ring Centre-of-Gravity numbers with atoms#
Cg(2) = Pt1-N2-N3-C20-C21
Cg(3) = N2-C10-C11-C12-C13-C21
Cg(4) = N3-C16-C17-C18-C19-C20
Cg(6) = C13-C14-C15-C16-C20-C21
Cg(7) = N2-C10-C11-C12-C13-C14-C15-C16-C20-C21
Cg(8) = N3-C13-C14-C15-C16-C17-C18-C19-C20-C21
Cg(9) = N2-N3-C10-C11-C12-C13-C14-C15-C16-C17-C18-C19-C20-C21
==
Analysis of X-H...Cg(Pi-Ring) Interactions (H..Cg < 3.0 Ang. - Gamma < 30.0 Deg)
==
- Cg(J) = Center of gravity of ring J (Plane number above)
- H-Perp = Perpendicular distance of H to ring plane J
- Gamma = Angle between Cg-H vector and ring J normal
- X-H..Cg = X-H-Cg angle (degrees)
- X..Cg = Distance of X to Cg (Angstrom)
- X-H, Pi = Angle of the X-H bond with the Pi-plane (i.e.' Perpendicular = 90 degrees, Parallel = 0 degrees)

X--H(I) Res(I) Cg(J) [ARU(J)]	H..Cg	H-Perp	Gamma	X-H..Cg	X..Cg	X-H,Pi
C(3) -H(3) [1] -> Cg(4) [2765.01]	2.86	2.77	14.67	124	3.484(2)	27
C(7) -H(7B) [1] -> Cg(5) [2665.01]	2.49	-2.49	2.16	160	3.437(2)	70

Min or Max 2.490 -2.488 2.2 160.00 3.437 70.00
[2765] = 2-X,1-Y,-Z
[2665] = 1-X,1-Y,-Z

Cg(4) = N3-C16-C17-C18-C19-C20
Cg(5) = C1-C2-C3-C4-C5-C6
==

Table S11. Selected bond distances and angles (Å, °) for **2**.

Pt1—C1	1.9970 (17)	C1—Pt1—N2	98.10 (7)
Pt1—N2	2.0264 (15)	C1—Pt1—N1	80.85 (7)
Pt1—N1	2.0859 (16)	N2—Pt1—N1	176.03 (6)
Pt1—N3	2.1512 (16)	C1—Pt1—N3	168.26 (6)
		N2—Pt1—N3	79.19 (6)
		N1—Pt1—N3	102.60 (6)

Table S12. Hydrogen bonding interactions (Å, °) for **2**.

**D—H···A**	**D—H**	**H···A**	**D···A**	**D—H···A**
O4—H4A···O2	0.85 (4)	1.95 (4)	2.779 (3)	163 (4)
O4—H4B···O3[i]	0.81 (4)	2.10 (4)	2.887 (3)	162 (4)
O5—H5A···O6[ii]	0.75 (4)	2.09 (4)	2.793 (3)	156 (4)
O5—H5B···O4	0.89 (4)	1.86 (4)	2.753 (3)	176 (4)
O6—H6A···O1	0.81 (5)	2.30 (5)	3.054 (3)	156 (4)
O6—H6A···O3	0.81 (5)	2.38 (5)	3.103 (4)	149 (4)
O6—H6B···O5[iii]	0.77 (5)	2.05 (5)	2.708 (3)	144 (5)

Symmetry codes: (i) $-x+1, -y, -z+1$; (ii) $x, y, z-1$; (iii) $-x, -y, -z+1$.

4. References:

(1) G. S. Nichol and W. Clegg, CrystEngComm **2007**, *9*, 959-960.

(2) J. W. Steed, *CrystEngComm* **2003**, *5*, 169-179.

(3) A. Gavezotti, *CrystEngComm* **2008**, *10*, 389-398.

(4) a) G. R. Desiraju, *CrystEngComm* **2007**, *9*, 91-92; b) J. Ruiz, V. Rodríguez, N. Cutillas, A. Hoffmann, A.-C. Chamayou, K. Kazmierczak, C. Janiak, *CrystEngComm* **2008**, *10*, 1928-1938.

(5) a) G. Althoff, J. Ruiz, V. Rodríguez, G. López, J. Pérez, C. Janiak, *CrystEngComm* **2006**, *8*, 662-665; b) X. Hao, S. Parkin, C. P. Brock, *Acta Crystallogr., Sect. B: Struct. Sci.* **2005**, *61*, 689-699; c) N. J. Babu, A. Nangia, *CrystEngComm* **2007**, *9*, 980-983; d) A. C. Chamayou, C. Biswas, A. Ghosh, C. Janiak, *Acta Cryst.* **2009**, *C65*, m311-m313; e) G. Makhloufi, K. Schütte, C. Janiak, *Z. Kristallogr. NCS* **2014**, *229*, 429-430.

(6) S. Roy, R. Banerjee, A. Nangia, G. J. Kruger, *Chem. Eur. J.* **2006**, *12*, 3777-3788.

(7) a) X.-J. Yang, F. Drepper, B. Wu, W.-H. Sun, W. Haehnel and C. Janiak, *Dalton Trans.* **2005**, 256-267 and Supplementary Material therein; b) C. Janiak, *J. Chem. Soc., Dalton Trans.* **2000**, 3885-3896.

(8) a) M. Nishio, *Phys. Chem. Chem. Phys.* **2011**, *13*, 13873-13900; b) M. Nishio, Y. Umezawa, K. Honda, S. Tsuboyama, H. Suezawa, *CrystEngComm* **2009**, *11*, 1757-1788; c) M. Nishio, *CrystEngComm* **2004**, *6*, 130-158; d) C. Janiak, S. Temizdemir, S. Dechert, W. Deck, F. Girgsdies, J. Heinze, M. J. Kolm, T. G. Scharmann, O. M. Zipffel, Eur. J. Inorg. Chem. **2000**, 1229-1241; e) Y. Umezawa, S. Tsuboyama, K. Honda, J. Uzawa, M. Nishio, *Bull. Chem. Soc. Jpn.* **1998**, *71*, 1207-1213; f) M. Nishio, M. Hirota and Y. Umezawa, The CH/π interaction (evidence, nature and consequences), Wiley-VCH, New York, **1998**.

(9) a) H. Hosseini Monfared, Z. Kalantari, M.-A. Kamyabi, C. Janiak, *Z. Anorg. Allg. Chem.* **2007**, *633*, 1945-1948; b) H. Masui, *Coord. Chem. Rev.* **2001**, *219-221*, 957-992; c) A. Castiñeiras, A. G. Sicilia-Zafra, J. M. Gonzáles-Pérez, D. Choquesillo-Lazarte, J. Niclós-Gutiérrez, *Inorg. Chem.* **2002**, *41*, 6956-6958; d) E. Craven, C. Zhang, C. Janiak, G. Rheinwald, H. Lang, *Z. Anorg. Allg. Chem.* **2003**, *629*, 2282-2290; e) C. Janiak, A.-C. Chamayou, A. K. M. R. Uddin, M. Uddin, K. S. Hagen, M. Enamullah, *Dalton Trans.* **2009**, 3698-3709.

(10) T. S. Piper, *J. Am. Chem. Soc.* **1961**, *83*, 3908-3909.

(11) a) A.-C. Chamayou, G. Makhloufi, L. A. Nafie, C. Janiak, S. Lüdeke, *Inorg. Chem.* **2015**, *54*, 2193-2203; b) A.-C. Chamayou, S. Lüdeke, V. Brecht, T. B. Freedman, L. A. Nafie, C. Janiak, *Inorg. Chem.* **2011**, *50*, 11363-11374; c) H. Sakiyama, H. Ōkawa, N. Matsumoto, S. Kida, *Bull. Chem. Soc. Jpn.* **1991**, *64*, 2644-2647; d) H. Sakiyama, H. Ōkawa, N. Matsumoto, S. Kida, *J. Chem. Soc., Dalton Trans.* **1990**, 2935- 2939; e) R. E. Ernst, M. J. O´Connor, R. H. Holm, *J. Am. Chem. Soc.* **1967**, *89*, 6104-6113.

(12) a) S. Hiroshi, O. Hisashi, M. Naohide, K. Sigeo, *Bull. Chem. Soc. Jpn.* **1991**, *64*, 2644-2647; b) A.-C. Chamayou, G. Makhloufi, L. A. Nafie, C. Janiak, S. Lüdeke, *Inorg. Chem.* **2015**, *54*, 2193-2203; c) A.-C. Chamayou, S. Lüdeke, V. Brecht, T. B. Freedman, L. A. Nafie, C. Janiak, *Inorg. Chem.* **2011**, *50*, 11363-11374

(13) a) A. Spek, *Acta Cryst. D* **2009**, *65*, 148-155; b) A. L. Spek, *PLATON - A multipurpose crystallographic tool, Utrecht University, Utrecht, The Netherlands, **2005***.

(14) a) V. Lozana, P.-G. Lassahn, C. Zhang, B. Wu, C. Janiak, G. Rheinwald, H. Lang, in *Z. Naturforsch. B,* **2003**, *58*, 1152-1164; b) C. Zhang, C. Janiak, *Z. Anorg. Allg. Chem.* **2001**, *627*, 1972-1975; c) C. Zhang, C. Janiak, *J. Chem. Crystallogr.* **2001**, *31*, 29-35; d) H.-P. Wu, C. Janiak, G. Rheinwald, H. Lang, *J. Chem. Soc., Dalton Trans.* **1999**, 183-190; e) C. Janiak, L. Uehlin, H.-P. Wu, P. Klufers, H. Piotrowski, T. G. Scharmann, *J. Chem. Soc., Dalton Trans.* **1999**, 3121-3131; f) H.-P. Wu, C. Janiak, L. Uehlin, P. Klufers, P. Mayer, *Chem. Commun.* **1998**, 2637-2638.

(15) a) N. N. Laxmi Madhavi, G. R. Desiraju, A. K. Katz, H. L. Carrell, A. Nangia, *Chem. Commun.* **1997**, 1953-1954; b) H.-C. Weiss, D. Blaser, R. Boese, B. M. Doughan, M. M. Haley, *Chem. Commun.* **1997**, 1703-1704; c) T. Steiner, M. Tamm, B. Lutz, J. Van Der Maas, *Chem. Commun.* **1996**, 1127-1128; d) P. L. Anelli, P. R. Ashton, R. Ballardini, V. Balzani, M. Delgado, M. T. Gandolfi, T. T. Goodnow, A. E. Kaifer, D. Philp, *J. Am. Chem. Soc.* **1992**, *114*, 193-218.
